# Extramedullary plasmacytoma: Tumor occurrence and therapeutic concepts—A follow‐up

**DOI:** 10.1002/cam4.4816

**Published:** 2022-05-16

**Authors:** Adrian Holler, Iwona Cicha, Markus Eckstein, Marlen Haderlein, Marina Pöttler, Anja Rappl, Heinrich Iro, Christoph Alexiou

**Affiliations:** ^1^ Department of Otorhinolaryngology, Head & Neck Surgery Universitätsklinikum Erlangen, Friedrich‐Alexander‐Universität Erlangen‐Nürnberg Erlangen Germany; ^2^ Department of Otorhinolaryngology, Head & Neck Surgery, Section of Experimental Oncology & Nanomedicine (SEON), Else Kröner‐Fresenius‐Stiftung‐Professorship, Universitätsklinikum Erlangen Friedrich‐Alexander‐Universität Erlangen‐Nürnberg Erlangen Germany; ^3^ Institute of Pathology University Hospital Erlangen, Friedrich‐Alexander‐Universität Erlangen‐Nürnberg Erlangen Germany; ^4^ Department of Radiation Oncology University Hospital, Friedrich‐Alexander‐Universität Erlangen‐Nürnberg Erlangen Germany; ^5^ Institut für Medizininformatik, Biometrie und Epidemiologie (IMBE) Friedrich‐Alexander‐Universität Erlangen‐Nürnberg Erlangen Germany

**Keywords:** extramedullary plasmacytoma, non‐Hodgkin lymphoma, survival, therapy, tumor outcomes

## Abstract

**Background:**

Extramedullary plasmacytoma (EMP) is a solitary tumor consisting of neoplastic plasma cells, with very little to no bone marrow involvement. EMPs are usually located in the head and neck region, but can also occur along the digestive tract, in lungs, or extremities.

**Methods:**

Following our publication on EMP, which appeared in 1999 (Cancer 85:2305–14), we conducted a literature search for EMP‐related reports published between 1999 and 2021. The documented cases, as well as 14 of our own patients from the ENT Clinic Erlangen, were extensively analyzed.

**Results:**

Between 1998 and 2021, 1134 patients with EMP were reported, for whom information about the tumor localization was available. Among those, 62.4% had EMP in the head and neck area and 37.6% in other body regions. Data on therapy were reported in 897 patients, including 34.3% who received radiation, 28.1% surgery, 22.6% a combination of surgery and radiation, and 15.9% another therapy. In 76.9% patients no recurrence or transformation to multiple myeloma (MM) was reported, 12.8% showed local recurrence and 10.2% developed MM. Radiotherapy alone was associated with a tendency for increased occurrence of MM. In patients with EMP of head and neck area, combination therapy (surgery and radiation) resulted in a 5‐year overall survival rate of 98.3%, surgery alone of 92.4%, and radiotherapy of 92.7%.

**Conclusions:**

Collectively, our analyses indicate that surgical resection alone can achieve long‐term tumor control in patients with EMP, if the tumor can be removed within safe limits without causing serious functional impairment. However, if this is not certain, either radiation or a combination of surgery and radiation therapy is suggested as an effective means of local tumor control.

## Graphical abstract

This paper provides an overview of the extramedullary plasmacytoma cases reported between 1999 and 2021. The global patients’ cohort and the current therapy options are compared to our previous report from 1999.

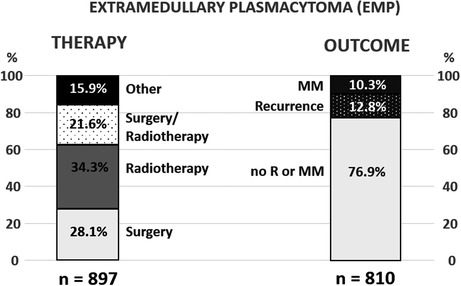

## 1 INTRODUCTION

Extramedullary plasmacytoma (EMP) belongs to the group of plasma cell neoplasms, which include following entities: multiple myeloma (MM), lymphoplasmacytic lymphoma, solitary plasmacytoma of the bone (SBP), and EMP.^1^ The EMP was first described by Schridde et al. in 1905.^2^ Since then, numerous articles including case reports were published on this relatively rare disease.

**FIGURE 1:**
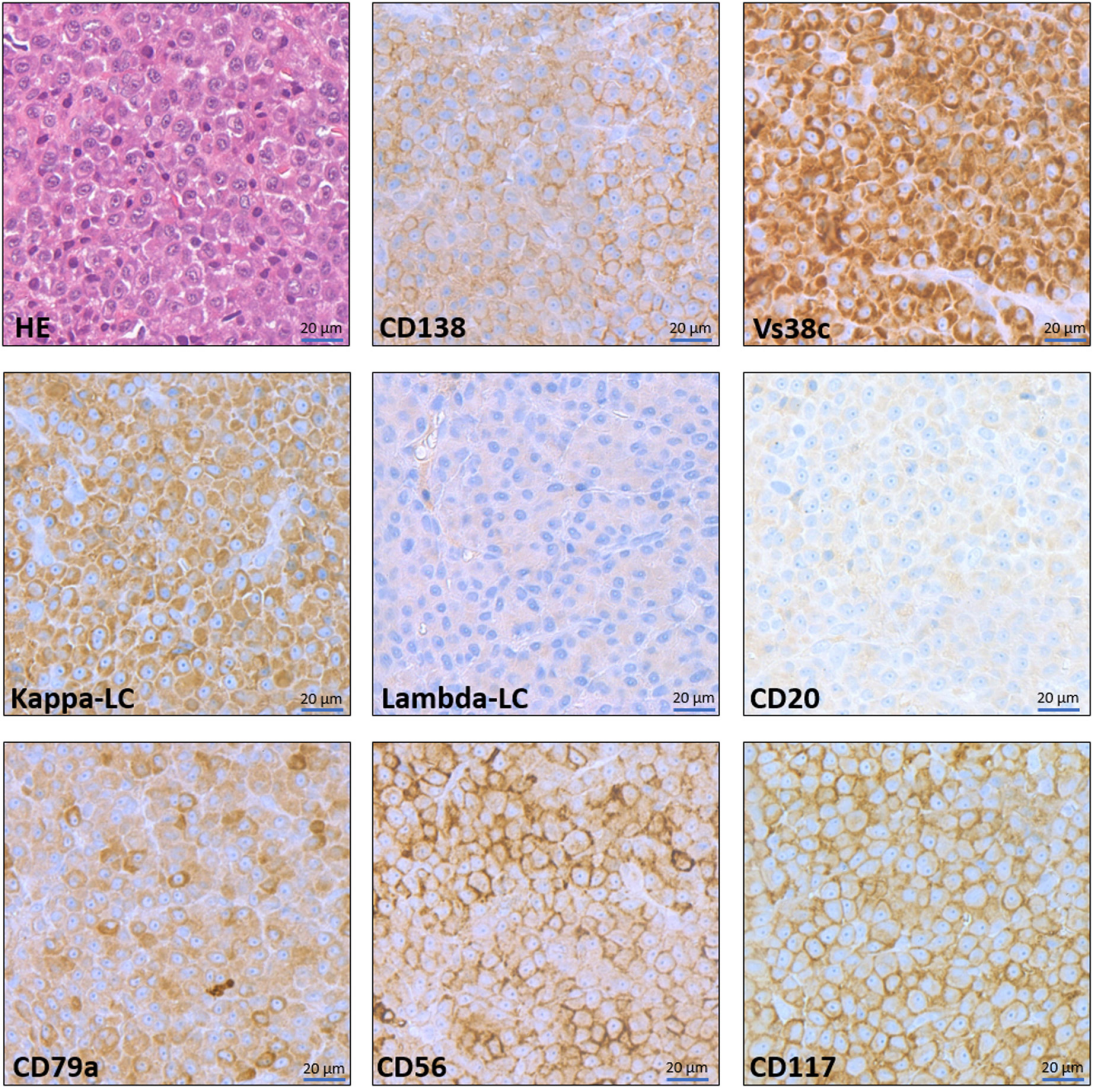
Representative images of an extramedullary plasmacytoma. The tumor cells show abundant basophilic cytoplasm, a small perinuclear “hof”, larger nuclei with remarkable nucleoli resembling immature plasma cells. Blastoid features are absent. Plasmacytoma cells express prototypical plasma cell markers (Vs38c, CD138) and show a light chain restriction (abundant expression of kappa light chains, completely negative for lambda light chains). Typically, CD20 is negative while the pan‐B‐cell/origin marker CD79a shows a specific positivity. Similar to plasma cell myelomas (multiple myeloma), the EMP cells show a strong aberrant CD56 and CD117 (c‐Kit) expression. All images were taken at 400x magnification from digitalized whole‐slide stainings, scale bar: 20 μm.

Due to the frequency of localization, a distinction is made between EMPs in the upper aero‐digestive tract (UAD) and those outside the UAD (non‐UAD). Furthermore, it can be distinguished between primary and secondary EMP, whereby the secondary EMP just describes an extramedullary manifestation of MM.^3^, ^4^ EMP is an immunoproliferative monoclonal disease of the B‐cell‐linage and originates from a transformed plasma cell, or from a clone of these cells. Plasma cells are highly differentiated and are able to synthesize immunoglobulins. The resulting tumor therefore homogenously expresses immunoglobulin, the increased levels of which can be found in blood sera or urine. In line with the WHO guidelines,^5^ EMP is commonly evaluated according to histological markers including Vs38c, CD138, kappa light chains (KLC), and lambda light chains (LLC), CD20, Cd79a, CD56, or CD117 (Figure 1). The EMP is generally a localized disease with 10–20% involvement of the lymph nodes.^6^ A conversion from EMP to MM is described in the literature, but the risk varies between 8% and 31%.^7^, ^8^, ^9^ To verify an EMP and, in particular, to distinguish EMP from MM, the following criteria must be fulfilled^1^, ^10^, ^11^: (1) Existence of one or more extramedullary plasma cell tumors; (2) Inconspicuous bone marrow smear with normal plasma cell ratio within the bone marrow, including inconspicuous plasma cell morphology, or less than 10% plasma cell ratio; (3) No radiological evidence of osteolysis; (4) No hypercalcemia or renal failure; (5) None or low M‐protein serum concentration. After tumor recurrence, the same criteria apply.

It is estimated that only about 3%–5% of plasma cell neoplasms are EMPs.^12^ Despite this fact, several hundred reports and case studies addressed EMP in the last 22 years. Following our publication on EMP from 1999,^13^ this study includes the documented cases from the literature published between 1998 and 2021 and a separate analysis of 14 of our own patients from the ENT Clinic Erlangen. The advantage of this approach is that it allows comparison of the patients' collectives reported before and after 1998, enabling observation of the trends in disease frequency and localization, therapy and outcomes.

## 2 PATIENTS AND METHODS

### 2.1 Literature research

An extensive literature search was carried out in the PUBMED/MEDLINE database, which identified all German and English language manuscripts on EMP published between 1998 and December 2021 (nearly 450 publications in total, for a complete list see Appendix S1). Following the publication of Alexiou et al. (1999), which considered all reports from 1905 (first description of the EMP) until 1997, this paper gives a complete overview of the EMP cases reported in the last 22 years.

Due to lack of information, such as the location of the tumor, as well as the fact that in many studies the patients could not be clearly assigned to individual parameters, not all cases could be evaluated with respect to each question. The given percentages therefore always refer only to the number of cases, which were documented in terms of the corresponding parameters.

### 2.2 Statistics

The predominant analysis was descriptive in nature. When proportions had to be tested against each other the Chi‐square test of independence was used. For event‐time‐analysis, the Kaplan and Meier method was employed, however, the prerequisites for the standard log‐rank‐test for differences in the survival of two or more groups were not fulfilled. Instead, point‐wise confidence intervals were calculated for differences in 5‐year‐survival for all pairwise group combinations respectively and checked for inclusion of zero.^14^ All patients discussed in the literature were included where data were available. Cases of missing data were excluded from the analysis (complete case analysis). The significance level for tests was set to 5%. The data were analyzed with the statistic software R version 4.0.3 (R Core Team, 2020).^15^

### 2.3 Erlangen cohort

Fourteen patients with EMP of the head and neck region were treated between 1998 and 2021 in the Otorhinolaryngology Clinic at the Universitätsklinikum Erlangen. These patients were diagnosed with EMP by fulfilling following criteria: (1) Presence of one or more extramedullary plasma cell tumors; (2) Inconspicuous bone marrow smears (normal plasma cell content in the bone marrow with inconspicuous plasma cell morphology or less than 10% plasma cell content); (3) No radiological evidence of osteolysis; (4) No hypercalcemia or kidney failure; (5) No or low serum M‐protein concentration.

## 3 RESULTS

### 3.1 Literature data analysis

The documented cases from the literature between 1998 and the end of 2021 were extensively analyzed. In total, the information on tumor localization was available in 1134 cases (Table 1). EMP may occur in every organ,^16^ but the predominant localization is in upper aero‐digestive (UAD), with 62.4% of EMPs (707 tumors) being detected in that area. About 37.6% EMPs, corresponding to 427 cases, were located in the non‐UAD body regions. In the UAD, 37.9% EMPs arise in the nasal cavity and paranasal sinus, 32.8% within the pharynx, 13.7% in the larynx (detailed localization of reported UAD cases is given in Table S1). In the non‐UAD area, locations of the EMP are more diversified and are listed in Table S2.

**TABLE 1:** Sites of EMP occurrence reported between 1998–2021

Site of occurrence: UAD	No. of cases	% of UAD
Nasal cavity or paranasal sinus	268	38
Pharynx	232	33
Larynx	97	14
Glands of the UAD	33	5
Other UAD	77	11

Site of occurrence: non‐UAD	No. of cases	% of non‐UAD
Gastrointestinal tract	131	31
Lung region	34	8
Urogenital tract	41	9
Other non‐UAD	221	52

*Note*: Total cases in the upper aerodigestive tract (UAD) *n* = 707, total cases in non‐UAD, *n* = 427. UAD cases represent 62% and non‐UAD cases 38% of the whole analyzed cohort (1134 cases). The percentages were rounded up to the whole numbers.

The gender of the patients was known in 984 cases (87% of all cases), 664 being male and 320 female. EMP in UAD‐regions was 2.3 times more likely to occur in men as in women (69.4%, *n* = 295 vs. 30.6%, *n* = 130), whereas the frequency ratio in non‐UAD areas was 1.4/1 for men versus women (58%, *n* = 196 vs. 42%, *n* = 142). The proportion differences in men and women for UAD‐ and non‐UAD‐regions are statistically significant (Chi‐square test, *p* = 0.004).

The age of the patients was documented in 768 cases (68% of analyzed cases), with mean age being 55.8 years (median 57 years, range: 3.5–95). EMPs develop more commonly in the second half of life, since the largest number of 434 documented cases in the UAD‐region have occurred in the sixth and seventh decade of life (*n* = 93 and *n* = 102, respectively). Similarly, the occurrence of EMP in non‐UAD regions (total 330 cases) was reported most commonly between the sixth and seventh decade of life (about 69–74 cases per decade).

### 3.2 Immunoglobulin status

The presence of KLC and LLC was reported in only 482 patients (43% of literature cases). Among those, 207 patients (42.9%) had LLC and 275 patients (57.1%) had KLC. Ig status was documented in 156 patients, whereby IgG was detected in 93 cases (59.6%), IgA in 39 cases (25%), IgM in 19 cases (12.2%) and IgD in five cases (3.2%). The presence of IgE was not observed. In UAD‐EMP, 96 patients with LLC (43.4%) and 125 with KLC (56.6%) were recorded and in non‐UAD, 86 patients with LLC (41.9%) and 119 with KLC (58.1%). This results in a ratio of LLC to KLC of 1:1.3 in UAD and 1:1.38 in non‐UAD. Ig status in UAD‐EMP patients was documented in 78 cases, with IgG presence in 42 cases (53.8%), IgA in 18 cases (23.1%), IgM in 15 cases (19.2%) and IgD in only three cases (3.8%). In patients with non‐UAD EMP, Ig status was reported in 59 cases, most common being IgG (61% *n* = 36), followed by IgA (30.5%, *n* = 18), IgM (5.1%, *n* = 3) and IgD 2 (3.4%, *n* = 2).

### 3.3 Therapy

The therapy was described in 897 patients (79% of literature cases), including radiotherapy alone (34.3%, *n* = 308), surgery alone (28.1%, *n* = 252), surgery with radiotherapy (21.6%, *n* = 194) and other therapies (15.9%, *n* = 143). There were no differences in the age or male/female ratio between patients undergoing the most common treatments (radiotherapy alone vs surgery alone). In patients treated with radiotherapy alone, significantly more patients had EMP of the UAD region (UAD to non‐UAD ratio of 3.1/1), while in the surgery group, nearly the same numbers of patients had EMP of UAD and non‐UAD (1.14/1).

**FIGURE 2:**
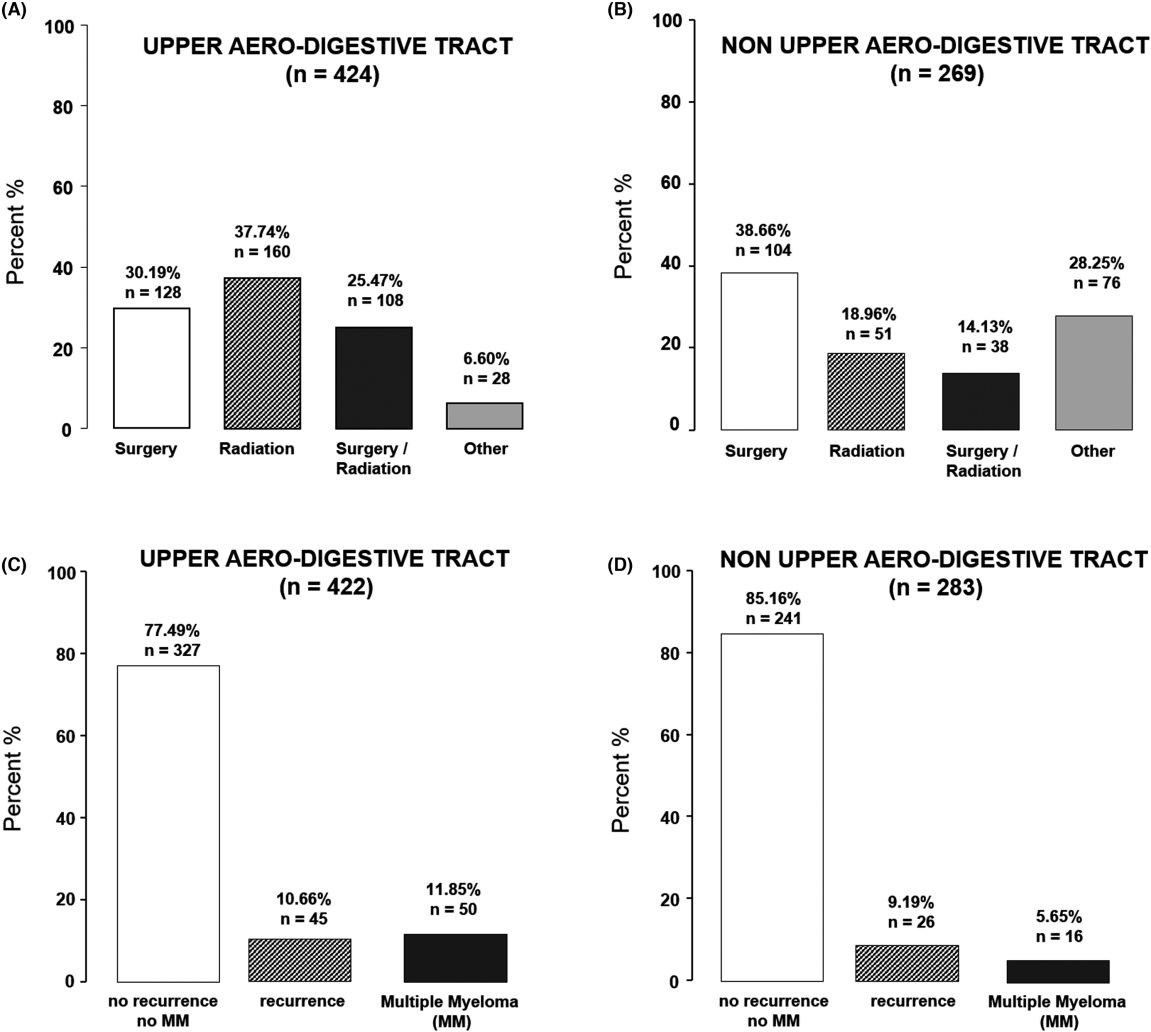
Most frequent treatment options (A, B) and overall progression (C, D) of extramedullary plasmacytoma (EMP) according to literature research. Other therapies in the UAD included chemotherapy (1.6% of EMP patients), radiation and chemotherapy (2.1%), surgery and chemotherapy (0.5%), surgery, chemotherapy and radiation (0.8%), and 2% patients received no therapy. In the non‐UAD area, chemotherapy was more common (16.2% of patients with EMP), followed by radiation and chemotherapy (4.9%), surgery and chemotherapy (4.9%), surgery, radiation and chemotherapy (1.2%), and 1.6% of patients received no therapy.

In 693 patients, both tumor localization and the form of therapy was described, among which 424 cases of EMP were in UAD and 269 cases developed in non‐UAD regions. The most common therapy for EMPs in the UAD‐area was radiation (37.7%, *n* = 160) followed by surgical intervention (30.2%, *n* = 128) and a combination of surgical intervention and radiation (25.5%, *n* = 108) (Figure 2A). Other forms of therapy, mainly consisting of chemotherapy alone, or in combination with radiation, or surgery or both, as well as no therapy, were rare (6.6%, *n* = 28). In the non‐UAD area, surgery was most often performed as a therapy (38.6%, *n* = 104) followed by other therapies (28.3%, *n* = 76), which include chemotherapy‐based therapies such as chemotherapy alone or in combination with radiation or surgery, or both. Radiation only therapy (19%, n = 51) as well as a combination of surgery and radiation (14.1%, *n* = 38) were less common in the non‐UAD area (Figure 2B).

With respect to the radiation dose in patients receiving radiotherapy alone, independent of tumor localization, the largest group of patients (*n* = 75) received doses in the range of 40–49 Gy. 62 patients received 50–59 Gy and 14 patients received 60 Gy or above. In 11 patients, doses below 40 Gy were administered. The data of the patients with known radiation regimen (*n* = 162) are summarized in Table S3, according to the radiation doses. Concerning the chemotherapy regimen, we found the detailed data in only 48 patients: In 14 cases, the therapy was based on cyclophosphamide with prednisone (in six patients in combination with vincristine, or vincristine and doxorubicin). Ten patients received VAD (vincristine, adriamycin, dexamethasone), and nine patients therapies based on bortezomib and dexamethasone (sometimes in combination with thalidomide, cyclophosphamide or doxorubicin). In eight patients, melphalan and prednisone was used and in two patients, VCMP (vincristine, cyclophosphamide, melphalan, prednisone) was used. Other patients received methotrexate (1), rituximab (1), rituximab with doxorubicin (1), dexamethasone (1), and thalidomide with dexamethasone (1).

### 3.4 Follow‐up and outcome

The duration of follow‐up was described in 568 patients (50% of literature cases), lasting on average 43.9 months (range 0.5–372 months). In six cases (followed up for an average 63.6 months), the assignment to UAD or non‐UAD was not possible. In 279 patients with EMP in UAD area, the average follow‐up period was 49.3 months, with average follow‐ups ranging between 49 and 55 months in patients treated with surgery alone, radiotherapy alone, or the combination of both. In patients receiving other types of therapy, the average follow‐up was 25.9 months. In 212 patients with EMP in non‐UAD area, the average follow‐up period was 35.1 months (range: 0.5–318). Recurrence‐free survival recorded in 505 patients was on average 35.7 months. In 273 analyzed patients with EMP in UAD area, average recurrence‐free survival was 39 months, and in 170 non‐UAD EMP patients, it was 25.1 months.

**FIGURE 3:**
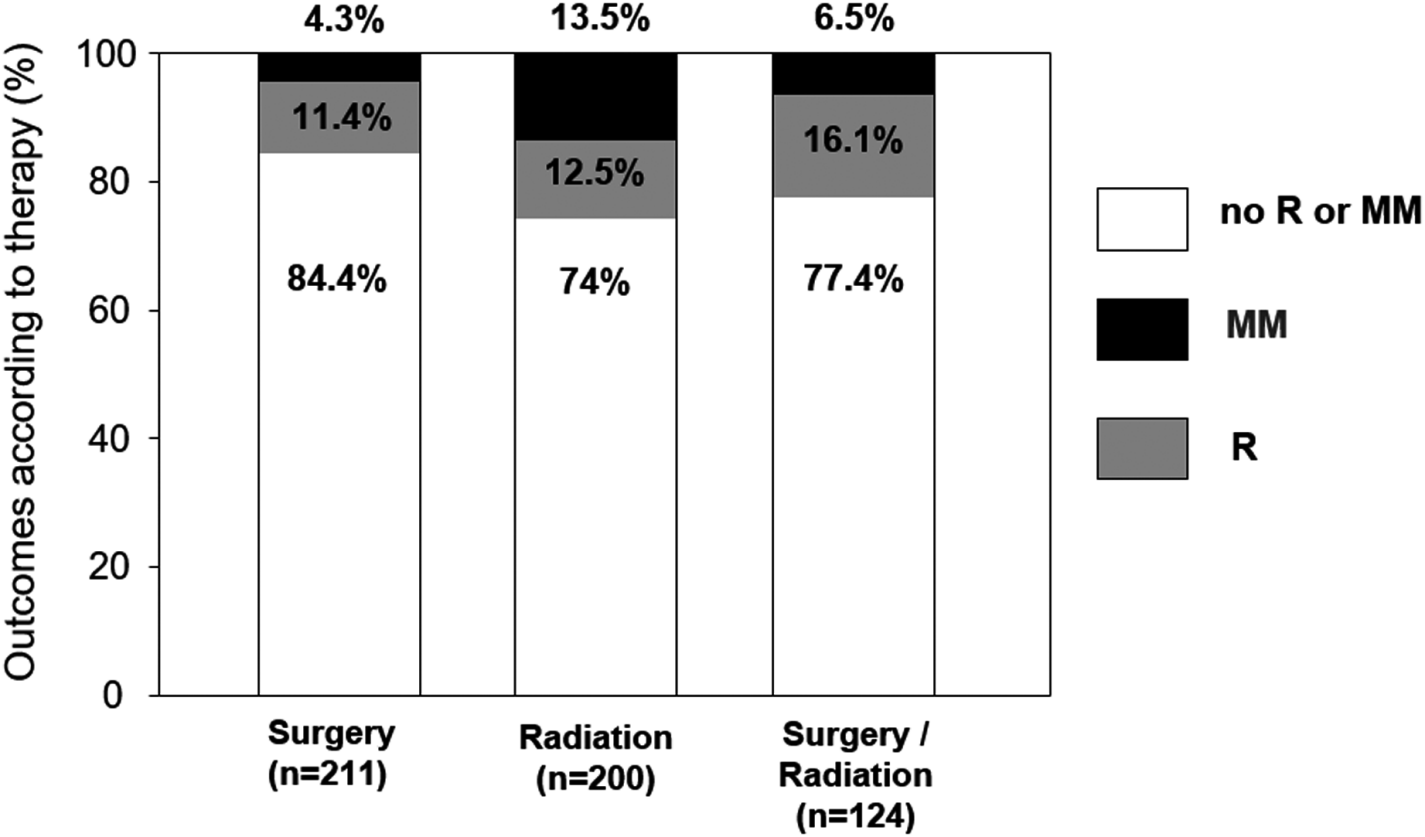
Outcomes in the patients with EMP dependent on therapy according to the literature search. Graph shows all patients for whom the outcomes were reported (*n* = 535), including those for whom no tumor localization was given. R, recurrence, MM, multiple myeloma.

Overall, the fate of 422 patients with UAD EMP and 283 patients with non‐UAD EMP was recorded. Among the UAD patients, the majority of cases (77.5%, *n* = 327) developed no MM or recurrence after treatment, whereas 10.7% (*n* = 45) had a recurrence and in 11.8% (*n* = 50), there was a conversion to MM (Figure 2C). However, when EMP was present in non‐UAD areas, 85.2% (*n* = 241) developed neither MM nor recurrence, in 9.2% patients (*n* = 26), there was recurrence and in 5.6% cases (*n* = 16), a conversion to MM was observed (Figure 2D). It was possible to correlate the fate of 646 patients with the data on their respective therapy. In 211 patients who received surgery alone, 84.4% (*n* = 178) had no recurrence and no MM. Recurrence was observed in 24 patients (11.4%) and MM in 9 (4.3%). Among 200 patients treated with radiotherapy alone, no recurrence and no MM was observed in 148 cases (74%), 25 patients (12.5%) had recurrence and 27 (13.5%) developed MM. This tendency for increased progression to MM in patients undergoing radiotherapy alone (Figure 3), was also clearly notable in the separate analysis of UAD and non‐UAD groups. In patients with EMP of the UAD, undergoing surgery alone, 5% developed MM, whereas in those treated with radiotherapy alone this percentage was increased nearly threefold, to 14%. In patients of the EMP of non‐UAD regions, the numbers were 3.4% versus 10.4% respectively. Among 124 patients treated with the combination of surgery and radiotherapy, 96 (77.4%) did not develop recurrence or MM, 20 (16.1%) developed recurrence and 8 (6.5%) had MM. In the remaining 111 patients treated with other therapies, 92 (82.8%) had no recurrence or MM, in 9 patients (8.1%) recurrence was observed and 10 patients (9%) developed MM.

### 3.5 Survival

**FIGURE 4:**
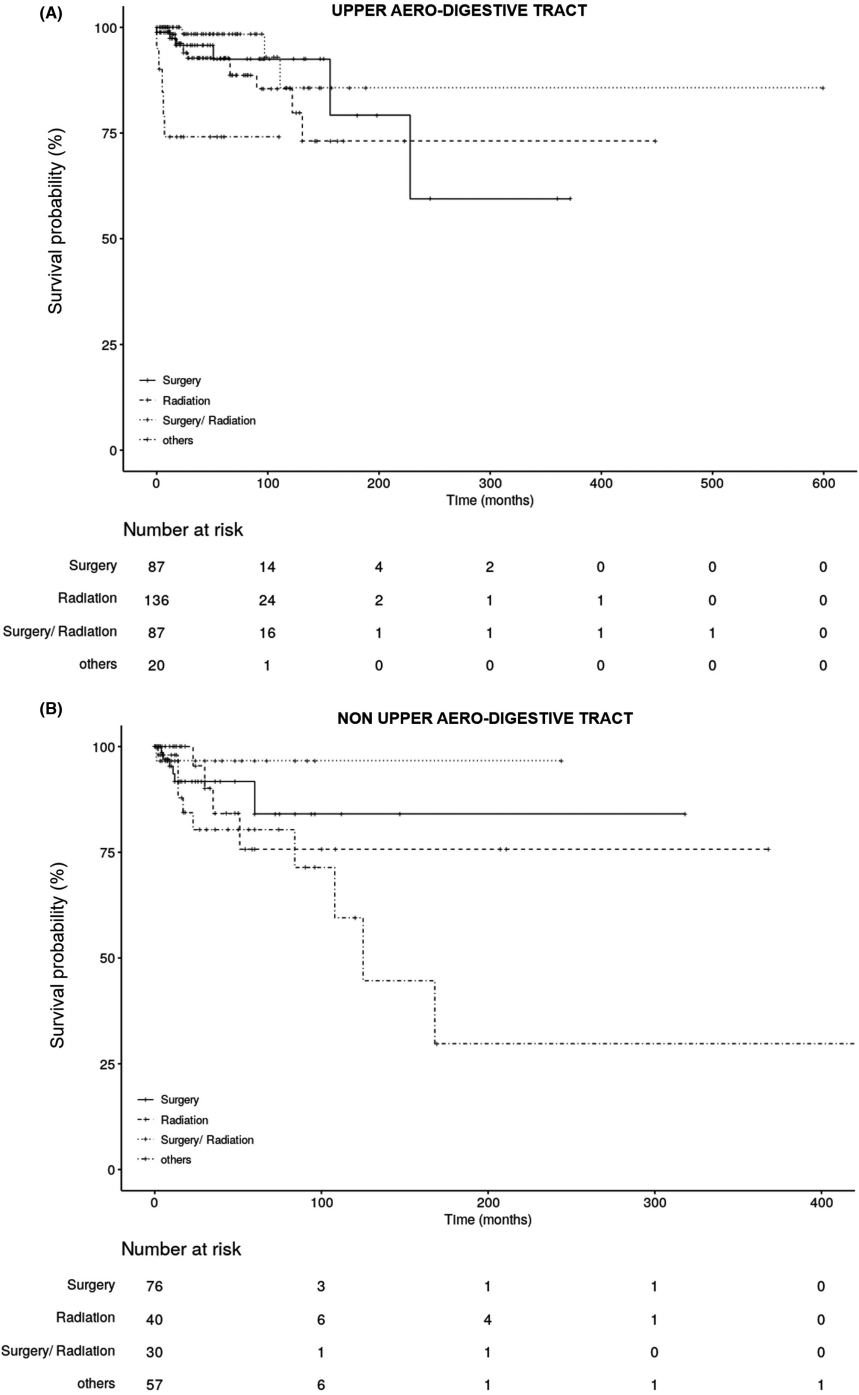
Overall survival curve (Kaplan–Meier) of patients with extramedullary plasmacytoma in (A) the upper aero‐digestive tract (UAD) and (B) outside of the upper aero‐digestive area (non‐UAD) compared to treatment according to the literature search.

The 5‐year‐overall survival probability of patients with EMP in UAD areas who had undergone combination therapy was 98.3% compared to patients who had received only surgery, or only radiation, where the survival rates were 92.4% and 92.7% respectively. For patients with other treatments, including chemotherapy‐based treatment, the 5‐year‐overall survival probability was 74.1% (Figure 4A). In patients with EMP in non‐UAD areas who had undergone combination therapy, the 5‐year‐overall survival of was also 96.7%. For those patients with surgical intervention alone, it was 84% and for the radiation‐treatment‐only patients it was 75.7%. The patients with non‐UAD EMP having received other therapies, comprising chemotherapy‐based interventions, had a 5‐year‐overall survival probability of 80.3% (Figure 4B). Since the prerequisites for the standard log‐rank‐test for differences in the survival of two or more groups were nonexistent, the point‐wise confidence intervals were calculated for differences in 5‐year‐survival for all pair‐wise combinations of therapies for UAD and non‐UAD respectively. Each confidence interval contained zero, thus no result was statistically significant.

### 3.6 Own cohort analysis

Additional separate analysis was performed on the Erlangen cohort diagnosed with EMP between 1999 and 2021 (see Table 2 and Figure 1). Among 14 own patients included in this study, nine patients were male and five patients were female with an average age of 65.5 years (median 71.5, range: 33 years–93 years). Due to the particular ENT clinic specialization, all EMPs were localized in the UAD tract. Five were located in the pharynx (three within the oropharynx and two in the nasopharynx); three at the nose sinuses, two in the larynx (glottis and supraglottis), and four in other UAD (one in parotid gland, one at the tonsils and two in the cervical lymph nodes of the UAD) (Table 2). Histological analysis of the tumors revealed that eight patients were positive for LLC, and four for KLC (two patients appeared with no histology). Seven patients were positive CD31, additionally six were positive for VS38c, four for CD79a, one for CD21 and one for CD117. One patient had an immunoglobulin (Ig)G and one patient had an IgG and IgA monoclonal by serum electrophoresis. Eight patients were treated with surgery alone, five had a combined radiation and surgery, whereas one patient received radiation therapy only. Doses of 46 to 60 Gy (median 56 Gy) were administered.

**TABLE 2:** Erlangen cohort: Patients' characterization, therapy, and follow‐up

Nr.	Age	Gender (m/f)	Localization	Light chains/Ig	Primary therapy	Outcome	Recurrence‐free survival (months)	Follo‐up (months)
1	77	m	Nasal cavity	kappa	S	DF (d)	204	204
2	46	f	Nasal cavity	kappa	S	DF	53	53
3	92	m	Oropharynx	lambda	S	Residual tumor at 20 m	20	20
4	79	f	Cervical lymph nodes	lambda	RT	Residual tumor, recurrence at 7 m (d)	7	12
5	50	m	Oropharynx	lambda	S	DF	7	7
6	33	m	Nasopharynx	kappa	S + RT	DF	4	65
7	57	m	Supraglottis area	kappa, IgG	S + RT	DF	70	70
8	80	m	Glottis area	lambda	S + RT	DF	53	53
9	55	m	Oropharynx	lambda	S	DF	190	190
10	55	m	Cervical lymph nodes	n.a.	S	DF	144	144
11	78	f	Nasopharynx	lambda	S	DF	202	202
12	93	f	Nasal cavity	lambda, IgG	S + RT	Recurrence at 4 m	4	94
13	66	f	Parotid gland	lambda	S + RT	Recurrence at 14 m (d)	14	29
14	79	m	Tonsils	n.a.	S	DF	48	48
Summary	median 71.5 (33–93)	m/f 9/5	5 pharynx3 nasal sinus2 larynx4 other UAD	kappa/lambda 4/8 (2 unknown)	8 S1 RT5 S + RT0 systemic	10 DF3 recurrences2 residual tumor	median 50.5 (4–204)	median 59 (7–204)

Abbreviations: d, dead; DF, disease‐free; m, month; n.a., not analyzed; S, surgery; RT, radiotherapy.

Patients were followed up for median of 59 months (range 7–204 months). The overall 5‐year survival rate for eight patients treated with surgery only was 100%, and for five patients treated with the combination therapy (radiation plus surgery) it was 80% (one death due to EMP at 29 months). In case of radiation only, the overall survival rate was 0% after 12 months, however, only one patient was treated solely with radiation. Furthermore, the recurrence occurred early in this patient, which may indicate incomplete tumor eradication or insufficient treatment. In *n* = 11 patients (78.6%) no recurrence and no transformation to a MM was detected (Table 2). Recurrence was observed in 21.4% (*n* = 3) patients. The median recurrence‐free survival was 50.5 months (range 4–204 months) and the median time of recurrence 7 months (range 4–14 months).

Several differences were noted upon comparison of our local cohort reported in 1999 with the present study cohort. The main differences include increased number of female patients, older age, as well as increased number of surgeries as a sole treatment option. In the 1999 cohort, the combination of surgery and radiotherapy was the preferred treatment option (five of seven cases), while among the patients recorded in the last 22 years, predominantly surgery alone was performed (8 of 14 cases).

## 4 DISCUSSION

Despite the rarity of the EMP,^12^ several hundred reports and case studies addressed this disease in the last 22 years. In this study, we focused on primary EMP and the literature cases of generalized disease or extramedullary manifestations of MM were excluded from the present analyses. Below, we discuss the differences noted in the global patients' cohort and the therapy options between 1999 and now. In comparison with the study published in 1999, more patients with a non‐UAD localization of EMP were reported during the last 22 years (427 cases vs. 155 reported between 1905–1998), indicating the improvement in differential diagnosis and a growing awareness of the fact that EMP may affect any body region.

### 4.1 Diagnosis

The first essential approach in the differential diagnosis of EMPs is the exclusion of other plasma cell diseases, including SBP, lymphoplasmacytic lymphoma, and especially of MM.^17^ As there are no groundbreaking radiologic differential diagnosis criteria,^18^ the disease must be verified histologically.^19^, ^20^, ^21^ The abnormal plasma cell of an EMP originates from a cell line and also produces the same Ig, mostly IgG KLC. Despite of this fact, the presence of KLC or LLC was mentioned in only 43% of literature cases. In the present analysis, the LLC to KLC ratio was 1:1.32 and was similar between patients with EMP of the UAD and non‐UAD. Interestingly, in Erlangen cohort, LLC were detected in most patients, so that the ratio LLC:KLC was 2:1, which is different than previously reported. Ig status was documented only in 156 literature patients (13.8% of the whole cohort), whereby IgG was detected most commonly, both in patients with EMP of the UAD and non‐UAD.

### 4.2 Treatment

The rarity of EMP and a long course of this disease make it difficult to determine the optimal treatment and prognostic parameters, which resulted in the lack of consensus on the best method to treat this condition.^6^, ^7^ Gonzalez‐Perez et al. described a rare case of spontaneous EMP remission without treatment.^22^ However, after exclusion of generalized disease, EMP should be treated as a locally aggressive and potentially metastatic tumor.^18^ Below, the specific treatment options and the differences noted concerning the therapy of the EMP with respect to our previous study are discussed.

#### 4.2.1 Surgery

If a tumor is easily accessible, resectable and has a low morbidity risk, surgical removal should be performed.^23^ Reducing tumor mass without a R0‐claim (no residual tumor remaining) may only be performed if it allows a rapid elimination of local symptoms (e.g., respiratory disability) and the effects of radiation therapy cannot be waited for. For most EMPs in the UAD region, surgery is not the best choice of treatment, as it may lead to serious mutilation.^17^, ^24^, ^25^, ^26^, ^27^, ^28^ In line with these considerations, the absolute numbers of surgeries as a sole treatment of EMP decreased in UAD regions, although this sole therapy approach remained among the three most commonly administered therapies in the UAD. In patients with EMP of non‐UAD areas, surgery was the main therapeutic option before 1999 (55.6%), but among the cases reported within the last 22 years, a shift toward other therapies was observed, in particular radiation and chemotherapy.

#### 4.2.2 Radiation

EMP is a highly radiosensitive tumor and the administration of appropriate therapy was reported to result in long‐term stability and potential healing in 67% of cases.^25^ According to previous reports, EMPs <5 cm have an excellent chance of local control by irradiation dose in the range of 40 Gy in 20 fractions, and the tumors >5 cm, with a higher risk of recurrence, should be treated with a higher dose in the 50 Gy range in 25 fractions.^8^, ^29^, ^30^, ^31^ Of note, the relatively scarce data available on tumor size and radiotherapy dose (Table S3) indicate that these guidelines are not always followed. Whereas among the 27 patients treated with 40–49 Gy, 18 had tumors smaller than 5 cm, only 6 of 20 recorded patients treated with doses of 50–59 Gy had tumors larger than 5 cm.

Previous reports indicated that local radiotherapy can reduce the risk of EMP recurrence to below 5%.^32^ However, an absolute assessment of success is only possible weeks after the completion of radiation therapy, because plasmacytomas respond slowly.^28^, ^33^ In those patients with EMP in the UAD area, in whom surgery may lead to serious mutilations and functional limitations, radiation therapy should be given preference, since no significant differences concerning recurrence‐free survival have been detected between these therapies (Figure 4A), neither in our analysis nor in previously published studies.^7^, ^34^, ^35^, ^36^ Our current analysis showed that recurrences were noted in 9.8% of patients with EMP of the UAD area treated solely with radiotherapy, which was slightly less than in patients who underwent surgery alone. However, radiotherapy as a sole treatment was associated with a tendency for increased occurrence of MM (14% vs. 5% in patients treated with surgery). Similar tendency with threefold increase in risk for MM development was observed in patients with EMP in non‐UAD areas, which confirms the trend observed in the analysis of the literature cases between 1905 and 1998.^13^ Interestingly, in comparison with that analysis, the absolute numbers of reported patients with EMP in the UAD treated only with radiation decreased by half, in parallel with the shift toward combination therapies. Despite this, radiation as the sole therapy still represents the most commonly selected treatment option in UAD (37.7% in the last 2 decades vs. 44.3% previously). In non‐UAD regions, more patients were treated only with radiotherapy during the last 20 years (19% vs. 11.1% previously), in parallel with the larger numbers of reported patients with EMP in non‐UAD areas.

Although radiation has been considered as first‐line therapy of EMP in the UAD,^37^ both surgery alone and a combination of surgery and radiation have achieved satisfactory results as shown in the present analysis and other studies.^29^ Generally, adjuvant radiation is not recommended for R0 surgery.^7^ However, if patients had been treated with primary surgery, but a residual tumor is confirmed (after R1 or R2 resection) and no further resection is considered, adjuvant radiotherapy should be performed.^31^, ^35^

Among patients with EMP of the UAD area, the absolute numbers of documented cases receiving the combination of surgery and radiotherapy were decreased in the last 22 years as compared with the older data (*n* = 108 vs. *n* = 176 previously), but percentually, the application of this combination therapy remained at the same level (25%–26%).

#### 4.2.3 Chemotherapy

In EMP, there is normally no indication for chemotherapy, even if local lymph nodes are involved.^17^, ^24^, ^38^ However, in case of large tumors, chemotherapy should be considered after radiotherapy, especially when the tumor is larger than 5 cm.^26^, ^39^ Furthermore, chemotherapy should be considered in case of a highly classified EMP according Bartl et al.^40^ and for refractory/persistent tumors, or recurrences.^31^ Our analysis showed that in non‐UAD regions, chemotherapy‐based therapies represented the second most often selected form of therapy (28.3%, *n* = 76), after the surgical removal (38.7%, *n* = 104). Chemotherapy only (16%) was more common than the combination of surgery and radiation (14.1%) in those patients. According to the analysis, patients receiving chemotherapy‐based therapies, including chemotherapy alone or in combination with radiation or surgery, or both, had a lower 5‐year‐overall survival probability as compared with those treated with surgery/radiation. This may be related to the fact that patients treated with systemic therapies, in particular chemotherapy, may be too frail for radiotherapy, or were affected by more advanced disease, thus necessitating more intensive treatment.

### 4.3 Aftercare, recurrence rate, and prognosis

Most studies have shown that solitary EMP forms have good to very good prognosis compared to other plasma cell tumors.^28^, ^36^ However, long‐term care is important because EMP patients bear a risk of developing MM or another disseminated cancer.^7^, ^17^, ^41^ From the localization point of view the proximity of bone tissue is important, since bone infiltration is a decisive factor for poor prognosis.^18^, ^42^ Also, a particular risk seems to be associated with tumors localized in the sinonasal tract: In the analysis of Bachar et al. 33% of patients, who died of local therapy failure and/or disease, had a tumor within the sinonasal tract.^7^ Other factors associated with poor prognosis and local high‐risk recurrence include tumor size (>5 cm), patient age (older than 60 years), incomplete therapy, and an initial increase in serum protein M.

In the present analysis, the majority of UAD patients (77.5%, *n* = 327) developed no MM or recurrence, whereas 10.7% (*n* = 45) had a recurrence and in 11.8% (*n* = 50), there was a conversion to MM. Among our 14 patients, three recurrences were detected, which represents a higher than average rate (21.4%), but no transition to MM was observed. In the analysis involving EMP of non‐UAD, only 9.2% patients had recurrence and the conversion to MM was observed in 6.7% cases. The occurrence of recurrences and MM conversions has markedly decreased compared to our analyses from 1999, where recurrences were reported in above 20% EMP cases, both in UAD and non‐UAD, which may reflect improved therapeutic approaches.

The limitation of this study is a low number of own patients with EMP and the fact that the analyses are retrospective. Although retrospective studies are very helpful in addressing diseases of low incidence, such as EMP, there is a risk of information bias, which may occur due to inaccurate records and incomplete information provided in the published reports.

## 5 CONCLUSIONS

In the follow‐up of our publication in 1999, we have attempted to record all EMP cases found in the UAD and other body areas that were published in the medical literature over the last 22 years. Of note, the number of cases reported in the non‐UAD regions markedly increased during that period. Concerning therapy, if an EMP is present in the soft tissue and is locally well‐operable, surgical resection alone is usually appropriate. Still, complete removal of an EMP is very often not feasible, especially in UAD, as close neighboring vital organ structures rule out radical surgery. For these patients, radiotherapy alone is recommended if the tumor is small. Overall, our analyses indicate that the combination therapy involving surgery and adjuvant radiotherapy seems to provide the best tumor control in patients with EMP in the UAD, but is also suitable if the disease is present outside UAD. However, the present analysis is a predominantly retrospective study, which is associated with a risk of information bias. Therefore, these results should be confirmed in a prospective multicenter randomized studies. Independent of the therapeutic regimen, patients diagnosed with a primary EMP should remain under lifelong medical observation, because even with successful primary therapy, either recurrences or a generalized plasmacytoma can occur years later.

## FUNDING INFORMATION

This work was supported by the Georg and Lou Zimmermann Stiftung, Thannhausen, Germany.

## CONFLICT OF INTEREST

The authors declare no conflict of interest.

## AUTHOR CONTRIBUTIONS

Adrian Holler: Investigation, data curation; writing – original draft; writing – review and editing. Iwona Cicha: Visualization; supervision; writing – original draft; writing – review and editing. Markus Eckstein: Visualization; writing – review and editing. Marlen Haderlein: Data curation; writing – review and editing. Marina Pöttler: Data curation; writing – original draft. Anja Rappl: Formal analysis; writing – original draft; writing – review and editing. Heinrich Iro: Resources; supervision; writing – review and editing. Christoph Alexiou: Conceptualization; supervision; writing – review and editing.

## INFORMED CONSENT

All patients treated at the Universitätsklinikum Erlangen have signed a general treatment contract, agreeing to the use of their data for scientific purposes in anonymized form.

## ETHICAL APPROVAL STATEMENT

The current study involving strictly retrospective analysis of anonymized data is exempt from ethical permission in line with the Basic law of the Federal Republic of Germany and the German Medical Association's professional code of conduct.

## Supporting information


Table S1
Table S2
Table S3

Appendix S1

## Data Availability

The data that support the findings of this study are available on request from the corresponding author. The data are not publicly available due to privacy or ethical restrictions.

## References

[1] Caers J , Paiva B , Zamagni E , et al. Diagnosis, treatment, and response assessment in solitary plasmacytoma: updated recommendations from a European expert panel. J Hematol Oncol. 2018;11(1):10. 29338789 10.1186/s13045-017-0549-1 PMC5771205

[2] Schridde H . Weitere Untersuchungen über die Körnelungen der Plasmazellen. Centralblatt f Allg Pathologie. 1905;16:433‐436.

[3] Sucker C , Stockschlader M . Extramedullary plasmacytoma. Dtsch Med Wochenschr. 2002;127(4):153‐155. 11807659 10.1055/s-2002-19696

[4] Rosinol L , Beksac M , Zamagni E , et al. Expert review on soft‐tissue plasmacytomas in multiple myeloma: definition, disease assessment and treatment considerations. Br J Haematol. 2021;194(3):496‐507. 33724461 10.1111/bjh.17338

[5] Swerdlow SH , Campo E , Harris NL WHO Classification of Tumours of Haematopoietic and Lymphoid Tissues (World Health Organization Classification of Tumours, Band 2). 2017.

[6] Susnerwala SS , Shanks JH , Banerjee SS , Scarffe JH , Farrington WT , Slevin NJ . Extramedullary plasmacytoma of the head and neck region: clinicopathological correlation in 25 cases. Br J Cancer. 1997;75(6):921‐927. 9062417 10.1038/bjc.1997.162 PMC2063399

[7] Bachar G , Goldstein D , Brown D , et al. Solitary extramedullary plasmacytoma of the head and neck‐long‐term outcome analysis of 68 cases. Head Neck‐J Sci Spec. 2008;30(8):1012‐1019. 10.1002/hed.20821 18327783

[8] Strojan P , Soba E , Lamovec J , Munda A . Extramedullary plasmacytoma: clinical and histopathologic study. Int J Radiat Oncol. 2002;53(3):692‐701. 10.1016/s0360-3016(02)02780-3 12062614

[9] Tournier‐Rangeard L , Lapeyre M , Graff‐Caillaud P , et al. Radiotherapy for solitary extramedullary plasmacytoma in the head‐and‐neck region: a dose greater than 45 Gy to the target volume improves the local control. Int J Radiat Oncol. 2006;64(4):1013‐1017. 10.1016/j.ijrobp.2005.09.019 16343803

[10] Galieni P , Cavo M , Pulsoni A , et al. Clinical outcome of extramedullary plasmacytoma. Haematologica. 2000;85(1):47‐51. 10629591

[11] Rajkumar SV , Dimopoulos MA , Palumbo A , et al. International myeloma working group updated criteria for the diagnosis of multiple myeloma. Lancet Oncol. 2014;15(12):e538‐e548. 25439696 10.1016/S1470-2045(14)70442-5

[12] Dimopoulos MA , Hamilos G . Solitary bone plasmacytoma and extramedullary plasmacytoma. Curr Treat Options Oncol. 2002;3(3):255‐259. 12057071 10.1007/s11864-002-0015-2

[13] Alexiou C , Kau RJ , Dietzfelbinger H , et al. Extramedullary plasmacytoma: tumor occurrence and therapeutic concepts. Cancer. 1999;85(11):2305‐2314. 10357398

[14] Kalbfleisch JD , Prentice RL . The statistical analysis of failure time data, 2. ed. Wiley Series in Probability and Statistics.2002.

[15] Team RC . A language and environment for statistical computing. Vienna, Austria:R Foundation for Statistical Computing. 2019.

[16] International Myeloma Working G . Criteria for the classification of monoclonal gammopathies, multiple myeloma and related disorders: a report of the international myeloma working group. Br J Haematol. 2003;121(5):749‐757. 12780789

[17] Markou K , Karasmanis I , Goudakos JK , Papaioannou M , Psifidis A , Vital V . Extramedullary plasmacytoma of temporal bone: report of 2 cases and review of literature. Am J Otolaryng. 2009;30(5):360‐365. 10.1016/j.amjoto.2008.07.004 19720260

[18] Baumann I , Ruck P , Dammann F , Plinkert PK . Locally recurring extramedullary plasmacytoma of the upper aerodigestive tract. Laryngorhinootologie. 2000;79(4):213‐220. 10838685 10.1055/s-2000-8800

[19] Zajko J , Czako L , Galis B . Plasmocytoma, multiple myeloma and plasma cell neoplasms in orofacial region. Bratisl Lek Listy. 2016;117(7):425‐427. 27546545 10.4149/bll_2016_083

[20] Agrawal SR , Chaudhary P , Rajput A , Jain AP . Pulmonary plasmacytoma with endobronchial extension: a rare presentation of solitary extramedullary plasmacytoma: a case report and brief review of literature. J Cancer Res Ther. 2015;11(4):1026. 10.4103/0973-1482.150350 26881595

[21] Alwan H , Moor JW , Wright D , Kanatas AN , Cruickshank HE . Extramedullary plasmacytoma of the tongue base: a case report and clinical review of head and neck plasmacytoma. Ent‐Ear Nose Throat. 2010;89(8):369. 20737375

[22] Gonzalez‐Perez LM , Borrero‐Martin JJ . An elderly man with a gingival mass that spontaneously regressed. Or Surg Or Med Or Pathol Or Radiol. 2016;121(4):348‐352. 10.1016/j.oooo.2015.08.014 26482192

[23] Huoh KC , Van Zante A , Eisele DW . Extramedullary plasmacytoma of the tonsil. Case Rep Otolaryngol 2011;2011:430809, 1, 2. 22937367 10.1155/2011/430809 PMC3420660

[24] Berrondo C , Gorman TE , Yap RL . Primary plasmacytoma of the testicle: a case report. J Med Case Reports. 2011;5:494. 10.1186/1752-1947-5-494 PMC3198716 21968163

[25] Weber DM . Solitary bone and extramedullary plasmacytoma. Hematology Am Soc Hematol Educ Program. 2005;2005(1):373‐376. 10.1182/asheducation-2005.1.373 16304406

[26] Aagre S , Madabhavi I , Patel A , Anand A , Panchal H , Parikh S . Primary Plurifocal extramedullary plasmacytoma of breast. Breast J. 2016;22(4):465‐466. 27059937 10.1111/tbj.12601

[27] Lee SH , Ahn BK , Baek SU , Chang HK . Primary isolated extramedullary plasmacytoma in the colon. Gastroenterology Res. 2013;6(4):152‐155. 27785246 10.4021/gr552w PMC5074814

[28] Menon S , Pujary K , Valiathan M . Dual pathology of the submandibular gland: plasmacytoma and pleomorphic adenoma. BMJ Case Rep. 2014;2014:bcr2013202463. 10.1136/bcr-2013-202463 PMC3948063 24591383

[29] Sasaki R , Yasuda K , Abe E , et al. Multi‐institutional analysis of solitary extramedullary plasmacytoma of the head and neck treated with curative radiotherapy. Int J Radiat Oncol Biol Phys. 2012;82(2):626‐634. 21277117 10.1016/j.ijrobp.2010.11.037

[30] Hotz MA , Schwaab G , Bosq J , Munck JN . Extramedullary solitary plasmacytoma of the head and neck. A clinicopathological study. Ann Otol Rhinol Laryngol. 1999;108(5):495‐500. 10335713 10.1177/000348949910800514

[31] Soutar R , Lucraft H , Jackson G , et al. Guidelines on the diagnosis and management of solitary plasmacytoma of bone and solitary extramedullary plasmacytoma. Br J Haematol. 2004;124(6):717‐726. 15009059 10.1111/j.1365-2141.2004.04834.x

[32] Smith E , Rottscholl R , Brosch S , Reiter R . Malignant lymphoma in the larynx. Laryngorhinootologie. 2013;92(6):381‐388. 23740416 10.1055/s-0033-1341465

[33] Naqash S , Sarmast AH , Showkat HI , et al. An unusual breast malignancy. Gulf J Oncolog. 2015;1(18):7‐9. 26003097

[34] Batsakis JG , Medeiros JL , Luna MA , El‐Naggar AK . Plasma cell dyscrasias and the head and neck. Ann Diagn Pathol. 2002;6(2):129‐140. 12004363 10.1053/adpa.2002.33458

[35] Antunes MI , Bujor L , Grillo IM . Anal canal plasmacytoma‐An uncommon presentation site. Rep Pract Oncol Radiother. 2010;16(1):36‐39. 24376953 10.1016/j.rpor.2010.12.002 PMC3863234

[36] Garelli M , Righini C , Faure C , Jankowski A , Brambilla C , Ferretti GR . Imaging of a case of extramedullary solitary plasmacytoma of the trachea. Case Rep Radiol 2011;2011:687203, 1, 4. 22606554 10.1155/2011/687203 PMC3350125

[37] Ghazizadeh M , Alavi Amlashi H , Mehrparvar G . Radioresistant extramedullary plasmacytoma of the maxillary sinus: a case report and review article. Iran J Otorhinolaryngol. 2015;27(81):313‐318. 26788481 PMC4710885

[38] Agarwal A . Neuroimaging of plasmacytoma. A Pictorial Review. Neuroradiol J. 2014;27(4):431‐437. 25196616 10.15274/NRJ-2014-10078 PMC4236876

[39] Kalan A , Asare‐Owusu L , Tariq M . Solitary extramedullary plasmacytoma of tonsil ‐ a rare location Indian. J Otolaryngol Head Neck Surg. 2000;52(3):285‐289. 10.1007/BF03006207 PMC3451112 23119699

[40] Bartl R , Frisch B , Fateh‐Moghadam A , Kettner G , Jaeger K , Sommerfeld W . Histologic classification and staging of multiple myeloma. A retrospective and prospective study of 674 cases. Am J Clin Pathol. 1987;87(3):342‐355. 3825999 10.1093/ajcp/87.3.342

[41] Bazaadut S , Soodin D , Singh P , et al. Extramedullary plasmacytoma of the tonsil with nodal involvement. Int J Otolaryngol. 2010;2010:302656. 20706681 10.1155/2010/302656 PMC2913787

[42] Zazpe I , Caballero C , Cabada T , Guerrero D , Gallo‐Ruiz A , Portillo E . Solitary thoracic intradural extramedullary plasmacytoma. Acta Neurochir. 2007;149(5):529‐532. 17404683 10.1007/s00701-007-1138-9

