# Supplementary material for: Extramedullary plasmacytoma: Tumor occurrence and therapeutic concepts—A follow‐up
Source: Cancer Med. 2022 May 16;11(24):4743–55. doi: 10.1002/cam4.4816 (PMC9761078; doi:10.1002/cam4.4816)
Supplement: Supplementary file 1 — Table S1 Table S2 Table S3 [file CAM4-11-4743-s001.docx]

**Supplementary information**

Extramedullary Plasmacytoma:

Tumor Occurrence and Therapeutic Concepts – a Follow-up

A. Holler, I. Cicha, M. Eckstein, M. Haderlein, M. Pöttler, A. Rappl, H. Iro, C. Alexiou

**Suppl. Table 1**: Sites of EMP occurrence reported in the upper aerodigestive tract (UAD) between 1998-2021. Total cases in the upper aerodigestive tract (UAD) n = 707. UAD cases represent 62% of the whole analyzed cohort (1134 cases).

| **Site of occurrence / UAD** | **No. of cases** | **% of UAD** |
| --- | --- | --- |
| **Nasal cavity or paranasal sinus** | **268** | **37.9** |
| Nasal cavity | 114 | 16.1 |
| Paranasal sinus | 40 | 5.7 |
| Maxillary sinus | 38 | 5.4 |
| Frontal sinus | 3 | 0.4 |
| Sphenoidal sinus | 12 | 1.7 |
| Ethmodial sinus | 9 | 1.3 |
| Nose area (unspecified) | 52 | 7.4 |
| **Pharynx** | **232** | **32.8** |
| Nasopharynx | 84 | 11.9 |
| Hypopharynx | 3 | 0.4 |
| Adenoids | 3 | 0.4 |
| Oropharynx | 115 | 16.3 |
| Tonsils | 7 | 1 |
| Pharynx area (unspecified) | 20 | 2.8 |
| **Larynx** | **97** | **13.7** |
| Supraglottis area | 16 | 2.3 |
| Subglottis area | 20 | 2.8 |
| Epiglottis area | 8 | 1.1 |
| Aryepiglotic folds | 1 | 0.1 |
| Glottis area | 20 | 2.8 |
| Sinus of Morgagni | 3 | 0.4 |
| Larynx area (unspecified) | 29 | 4.1 |
| **Glands of the UAD** | **33** | **4.7** |
| Parodis gland | 12 | 1.7 |
| Submandibular gland | 4 | 0.6 |
| Thyroid gland | 17 | 2.4 |
| **Other UAD** | **77** | **10.9** |
| Lymph nodes | 7 | 1 |
| Esophagus | 5 | 0.7 |
| Trachea | 9 | 1.3 |
| Lacrimal gland | 3 | 0.4 |
| Ear | 1 | 0.1 |
| Mandibular region | 1 | 0.1 |
| Skin | 2 | 0.3 |
| Other UAD (unspecified) | 49 | 7.5 |

**Suppl. Table 2**. Sites of EMP occurrence recorded in the non-upper aero-digestive tract (non-UAD). Total cases in non-UAD, n= 427. Non-UAD cases represent 38% of the whole analyzed cohort (1134 cases).

| **Site of occurrence / non-UAD** | **No. of cases** | **% of non-UAD** |
| --- | --- | --- |
| **Gastrointestinal tract** | **131** | **30.7** |
| Stomach | 46 | 10.8 |
| Duodenum | 17 | 4 |
| Colon | 16 | 3.8 |
| Rectum | 8 | 1.9 |
| Caecum | 7 | 1.6 |
| Ileum | 6 | 1.4 |
| Pancreas | 7 | 1.6 |
| Jejunum | 5 | 1.2 |
| Liver | 8 | 1.9 |
| Intestines | 2 | 0.5 |
| Small intestine | 5 | 1.2 |
| Large intestine | 2 | 0.5 |
| Gallbladder | 1 | 0.2 |
| Esophagus | 1 | 0.2 |
| **Lung region** | **34** | **7.9** |
| Lungs | 24 | 5.6 |
| Bronchi | 7 | 1.6 |
| Pleura | 3 | 0.7 |
| **Urogenital tract** | **41** | **9.6** |
| Ovary | 3 | 0.7 |
| Uterus | 3 | 0.1 |
| Penis | 2 | 0.5 |
| Vesico-uterine pouch | 4 | 0.9 |
| Testis | 10 | 2.3 |
| Kidney | 14 | 3.3 |
| Ureter | 3 | 0.7 |
| Urethra | 2 | 0.5 |
| **Other non-UAD** | **221** | **51.8** |
| Skin | 58 | 13.6 |
| Dura mater | 20 | 4.7 |
| Retroperitoneal space | 29 | 7 |
| Lymph nodes | 26 | 6.1 |
| Breast | 14 | 3.1 |
| Mediastinum | 12 | 2.8 |
| Brain | 6 | 1.4 |
| Musculature | 6 | 1.4 |
| Eye | 4 | 0.9 |
| Retina | 2 | 0.5 |
| Mesentery | 5 | 1.2 |
| Heart | 4 | 0.9 |
| Conjunctiva | 3 | 0.7 |
| Extrapleural tissue | 1 | 0.2 |
| Knee | 1 | 0.2 |
| Temple | 1 | 0.2 |
| Clivus | 1 | 0.2 |
| Spinal cord | 1 | 0.2 |
| Spleen | 1 | 0.2 |
| Sella turcica | 3 | 0.7 |
| Spinal canal | 1 | 0.2 |
| Soft tissue | 2 | 0.5 |
| Inguinal region | 1 | 0.2 |
| Unspecified non-UAD | 18 | 4.6 |

**Suppl. Table 3**. Comparison of patients with EMP treated with radiotherapy alone according to the radiation dose. Total cases with known therapy regimen, n= 162.

| **Radiation dose** | **27 - 39 Gy** | **40 - 49 Gy** | **50 - 59 Gy** | **60 - 70 Gy** |
| --- | --- | --- | --- | --- |
| Number of patients (% of total) | 11 (6.8%) | 75 (46.3%) | 62 (38.3%) | 14 (8.6%) |
| Male/female (ratio m/f) n (%) | 5/3 (1.7/1)  n=8 (73%) | 41/23 (1.8/1)  n=64 (85%) | 37/16 (2.3/1)  n=53 (85%) | 12/2 (6/1)  n=14 (100%) |
| Age (years), median (range) | 62.5 (25-76)  n=8 (73%) | 55 (20-86)  n=64 (85%) | 56.5 (16-87)  n=50 (81%) | 61.5 (25-76)  n=14 (100%) |
| UAD/non-UAD, n (%) | 5/5  n=10 (91%) | 54/21  n=75 (100%) | 53/9  n=62 (100%) | 13/1  n=14 (100%) |
| Tumor size <5 cm / >5 cm, n (%) | 5/0  n=5 (45%) | 18/9  n=27 (36%) | 14/6  n=20 (32%) | 1/0  n=1 (7%) |
| Outcome data, n (%) | 8 (73%) | 66 (88%) | 55 (89%) | 13 (93%) |
| Recurrence, n (%) | 0 | 8 (12.1%) | 5 (9.1%) | 1 (7.7%) |
| MM, n (%) | 1 (12.5%) | 10 (15.1%) | 8 (14.5%) | 0 |
| Residual tumor, n (%) | 0 | 11 (16.7%) | 10 (18.2%) | 1 (7.7%) |
| Disease-free time (months), median/mean | 15/33 | 12/39 | 26/43 | 50/85 |

MM, multiple myeloma; UAD, upper aero-digestive tract
