# Supplementary material for: Extramedullary plasmacytoma: Tumor occurrence and therapeutic concepts—A follow‐up
Source: Cancer Med. 2022 May 16;11(24):4743–55. doi: 10.1002/cam4.4816 (PMC9761078; doi:10.1002/cam4.4816)
Supplement: Supplementary file 2 — Appendix S1 [file CAM4-11-4743-s002.docx]

**Holler et al.**

**List of manuscripts published between 1999 and 2021 used for the literature data analysis:**

1. Aagre, S., et al. (2016). "Primary Plurifocal Extramedullary Plasmacytoma of Breast." Breast J **22**(4): 465-466.
2. Abrar, S., et al. (2018). "Extramedullary plasmacytoma: rare neoplasm of parotid gland." BMJ Case Rep **2018:bcr2017222367**.
3. Abrunhosa-Branquinho, A., et al. (2015). "A case of post-radiotherapy gastritis: radiation does not explain everything." Case Rep Oncol **8**(1): 9-14.
4. Agrawal, S. R., et al. (2015). "Pulmonary plasmacytoma with endobronchial extension: A rare presentation of solitary extramedullary plasmacytoma: A case report and brief review of literature." J Cancer Res Ther **11**(4): 1026.
5. Agreda Moreno, B., et al. (2013). "Extramedullary plasmacytoma as an exceptional location in frontal sinus." Acta Otorrinolaringol Esp **64**(5): 374-376.
6. Ahamed, E., et al. (2003). "Extramedullary plasmacytoma of the eyelid." Br J Ophthalmol **87**(2): 244-245.
7. Ahmed, A. R., et al. (2000). "Extramedullary plasmacytoma presenting with myasthenia gravis and mediastinal mass." Ann Thorac Surg **70**(4): 1390-1392.
8. Ahmed, M., et al. (2009). "Autologous bone marrow transplanation for extramedullary plasmacytoma presenting as adrenal incidentaloma." Ann Saudi Med **29**(3): 219-222.
9. Ahnach, M., et al. (2013). "Extramedullary plasmocytoma relapsing at differents sites: an unusual presentation." Pan Afr Med J **14**: 34.
10. Alabed, Y. Z., et al. (2014). "Solitary extramedullary plasmacytoma of the parotid gland imaged with 18F-FDG PET/CT." Clin Nucl Med **39**(6): 549-550.
11. Albano, D., et al. (2018). "18F-FDG PET/CT in solitary plasmacytoma: metabolic behavior and progression to multiple myeloma." Eur J Nucl Med Mol Imaging **45**(1): 77-84.
12. Alwan, H., et al. (2010). "Extramedullary plasmacytoma of the tongue base: a case report and clinical review of head and neck plasmacytoma." Ear Nose Throat J **89**(8): 369-373.
13. Ammar, T., et al. (2010). "Primary antral duodenal extramedullary plasmacytoma presenting with melena." Clin Gastroenterol Hepatol **8**(1): A32.
14. Ampil, F. L., et al. (2002). "Cavernous sinus involvement by extramedullary plasmacytoma of the sphenoid sinus. An argument for the use of adjuvant chemotherapy." Leuk Lymphoma **43**(10): 2037-2040.
15. Andrea, R., et al. (2013). "Primary extramedullary plasmacytoma of the heart: a rare manifestation of plasmacellular tumor." Case Rep Radiol **2013**: 290849.
16. Antunes, M. I., et al. (2010). "Anal canal plasmacytoma-An uncommon presentation site." Rep Pract Oncol Radiother **16**(1): 36-39.
17. Araujo Rde, P., et al. (2008). "Rare nasosinusal tumors: case series and literature review." Braz J Otorhinolaryngol **74**(2): 307-314.
18. Ashraf, M. J., et al. (2013). "Extramedullary plasmacytoma of the nasal cavity report of three cases with review of the literature." Iran Red Crescent Med J **15**(4): 363-366.
19. Attanasio, G., et al. (2006). "Extramedullary plasmacytoma of paranasal sinuses. A combined therapeutic strategy." Acta Otorhinolaryngol Ital **26**(2): 118-120; discussion 120.
20. Avila, A., et al. (2009). "Clinical features and differential diagnoses of solitary extramedullary plasmacytoma of the thyroid: a case report." Ann Diagn Pathol **13**(2): 119-123.
21. Bachar, G., et al. (2008). "Solitary extramedullary plasmacytoma of the head and neck--long-term outcome analysis of 68 cases." Head Neck **30**(8): 1012-1019.
22. Baek, B. J., et al. (2005). "Extramedullary plasmacytoma arising from the nasal septum." Ear Nose Throat J **84**(11): 720-722.
23. Baghmar, S., et al. (2013). "Solitary plasmacytoma: 10 years' experience at All India Institute of Medical Sciences, New Delhi." Leuk Lymphoma **54**(8): 1665-1670.
24. Basavaiah, S.H., et al., (2019). ”Clinicopathological spectrum of solitary Plasmacytoma: a single center experience from coastal India”. BMC Cancer **19**(1):801.
25. Baumann, I., et al. (2000). "[Locally recurring extramedullary plasmacytoma of the upper aerodigestive tract]." Laryngorhinootologie **79**(4): 213-220.
26. Bazaadut, S., et al. (2010). "Extramedullary plasmacytoma of the tonsil with nodal involvement." Int J Otolaryngol **2010**.
27. Belic, B., et al. (2013). "Nasal septum extramedullary plasmacytoma." Vojnosanit Pregl **70**(2): 221-224.
28. Berrondo, C., et al. (2011). "Primary plasmacytoma of the testicle: a case report." J Med Case Rep **5**: 494.
29. Bhadauria, M., et al. (2014). "Primary orbital plasmacytoma mimicking lacrimal gland tumor." Orbit **33**(4): 305-307.
30. Bhangoo, R.S., et al., (2021). “Obstructive rectosigmoid colon solitary extramedullary plasmacytoma
31. Dig Liver Dis 53(4):496-497.
32. Bhat, R. V., et al. (2010). "Plasmacytoma of tonsil diagnosed by fine-needle aspiration cytology." J Cytol **27**(3): 102-103.
33. Bhat, V., et al. (2014). "Extramedullary plasmacytoma of thyroid - a mimicker of medullary carcinoma at fine needle aspiration cytology: A case report." J Cytol **31**(1): 53-56.
34. Bidros, M., et al. (2011). "High-grade solitary extramedullary plasmacytoma arising in skeletal muscle of a kidney transplant recipient." Leuk Res **35**(9): e181-183.
35. Bila, J., et al. (2013). "Extramedullary plasmacytoma of the tongue base: a rare presentation of head and neck plasmacytoma." Vojnosanit Pregl **70**(10): 972-975.
36. Binnani, P., et al. (2010). "Disease recurrence in a transplant kidney in a patient with extramedullary plasmacytoma." Indian J Med Paediatr Oncol **31**(3): 91-93.
37. Bizzaro, N. and F. Fiorin (1999). "Coexistence of erythrocyte agglutination and EDTA-dependent platelet clumping in a patient with thymoma and plasmocytoma." Arch Pathol Lab Med **123**(2): 159-162.
38. Boll, M., et al. (2010). "Extramedullary plasmacytoma are characterized by a 'myeloma-like' immunophenotype and genotype and occult bone marrow involvement." Br J Haematol **151**(5): 525-527.
39. Bourtsos, E. P., et al. (2000). "Thyroid plasmacytoma mimicking medullary carcinoma: a potential pitfall in aspiration cytology." Diagn Cytopathol **23**(5): 354-358.
40. Brandt, H.H., et al., (2020). “Solitary extramedullary plasmacytoma of the larynx: a rare cause of dysphonia”. BMJ Case Rep **13**(9):e234478.
41. Bruns, F., et al. (2010). "Extramedullary plasmocytoma: a rare case with bifocal manifestation at uncommon sites." Anticancer Res **30**(5): 1779-1781.
42. Bucca, C., et al. (2009). "Severe episodic dyspnoea and abnormal flow-volume loop. Extramedullary plasmacytoma (EMP)." Thorax **64**(3): 210, 264.
43. Burgos-Blasco, B., et al., (2020). “Extramedullary plasmacytoma of the eyelid”. J Fr Ophtalmol **43**(2):177-178.
44. Burns, J. A., et al. (1999). "Extensive extramedullary amyloid-rich plasmacytoma of the mandible." Otolaryngol Head Neck Surg **120**(6): 937-939.
45. Cangiarella, J., et al. (2000). "Plasmacytoma of the breast. A report of two cases diagnosed by aspiration biopsy." Acta Cytol **44**(1): 91-94.
46. Cantone, E., et al. (2017). "Cyberknife® system: a new therapeutic strategy for sinonasal solitary extramedullary plasmacytomae." J Biol Regul Homeost Agents **31**(3): 763-768.
47. Cantone, E., et al. (2017). "Strategy for the treatment and follow-up of sinonasal solitary extramedullary plasmacytoma: a case series." J Med Case Rep **11**(1): 219.
48. Cao, D., et al. (2016). "Retroperitoneal laparoscopic management of a solitary extramedullary plasmacytoma associated with human immunodeficiency virus infection: A case report." Oncol Lett **11**(1): 767-769.
49. Cao, D., et al. (2014). "Solitary extramedullary plasmacytoma of the adrenal gland: a rare case report with review of the literature." Int J Clin Exp Pathol **7**(12): 9072-9075.
50. Carneiro, F. P., et al. (2009). "Extramedullary plasmocytoma associated with a massive deposit of amyloid in the duodenum." World J Gastroenterol **15**(28): 3565-3568.
51. Carrinola, R., et al., (2020). “Tracheal extramedullary plasmacytoma: a rare cause of chronic cough”. Monaldi Arch Chest Dis **90**(4).
52. Celebi, I., et al. (2018). "Tonsillar Plasmacytoma: clues on magnetic resonance imaging." BMC Med Imaging **18**(1): 19.
53. Celik, G., et al. (2010). "Bronchoscopic electrocautery therapy of a solitary endobronchial extramedullary plasmacytoma." Tuberk Toraks **58**(4): 435-438.
54. Cesana, C., et al. (2002). "Prognostic factors for malignant transformation in monoclonal gammopathy of undetermined significance and smoldering multiple myeloma." J Clin Oncol **20**(6): 1625-1634.
55. Chalazonitis, A. N., et al. (2005). "Extramedullary plasmacytoma of the sphenoid sinus." JBR-BTR **88**(6): 343.
56. Chang, Y. L., et al. (2014). "Extramedullary plasmacytoma of the nasopharynx: A case report and review of the literature." Oncol Lett **7**(2): 458-460.
57. Chao, M. W., et al. (2005). "Radiotherapy in the management of solitary extramedullary plasmacytoma." Intern Med J **35**(4): 211-215.
58. Chen, K. Y., et al. (1998). "Primary pulmonary plasmacytoma with lobar consolidation: an unusual presentation." J Formos Med Assoc **97**(7): 507-510.
59. Chen, T. C., et al. (1998). "Solitary extramedullary plasmacytoma in the retroperitoneum." Am J Hematol **58**(3): 235-238.
60. Chetty, R., et al. (2003). "Primary extramedullary plasmacytoma of the esophagus." Ann Diagn Pathol **7**(3): 174-179.
61. Chim, C. S., et al. (2005). "Plasma cell problems: Case 2. Extramedullary cardiac plasmacytoma presenting with cardiac tamponade." J Clin Oncol **23**(13): 3140-3143.
62. Chim, C. S., et al. (2001). "Primary extramedullary plasmacytoma of the lacrimal gland." Leuk Lymphoma **42**(4): 831-834.
63. Ching, A. S., et al. (2002). "CT and MR imaging of solitary extramedullary plasmacytoma of the nasal tract." AJNR Am J Neuroradiol **23**(10): 1632-1636.
64. Cobzeanu, M. D., et al. (2000). "Therapeutical aspects of the plasmocytoma localized at the level of head and neck." Rev Med Chir Soc Med Nat Iasi **104**(2): 151-154.
65. Coelho, L. R., et al. (2015). "Extramedullary plasmacytoma in the right pulmonary hilum." Radiol Bras **48**(6): 401-402.
66. Comba, I. Y., et al., (2019). “A Rare Case of Extramedullary Plasmacytoma Presenting as Massive Upper Gastrointestinal Bleeding”. Cureus **11**(1):e3993.
67. Comfere, N. I., et al. (2013). "Cutaneous extramedullary plasmacytoma: clinical, prognostic, and interphase cytogenetic analysis." Am J Dermatopathol **35**(3): 357-363.
68. Corvo, M. A., et al. (2013). "Extramedullary nasal plasmacytoma: Literature review and a rare case report." Int Arch Otorhinolaryngol **17**(2): 213-217.
69. Courtmans, I., et al. (2000). "Upper airways locations of plasmocytoma." Acta Otorhinolaryngol Belg **54**(4): 487-490.
70. Creach, K. M., et al. (2009). "Radiotherapy for extramedullary plasmacytoma of the head and neck." Int J Radiat Oncol Biol Phys **73**(3): 789-794.
71. Csomor, J., et al. (2018). "Extramedullary Plasmacytoma of the Pancreas Complicated with Left-Sided Portal Hypertension-a Case Report and Literature Review." J Gastrointest Cancer.
72. Cunovic, N., et al., (2020). “A Case Report of Solitary Extramedullary Plasmacytoma of the Cricoid Cartilage Diagnosed After Total Thyroidectomy”. Ear Nose Throat J **99**(2):130-131.
73. Dagan, R., et al. (2009). "Solitary plasmacytoma." Am J Clin Oncol **32**(6): 612-617.
74. de Camargo Moraes, P., et al. (2016). "Extramedullary Plasmacytoma Diagnosed in an HIV-Positive Patient by an Unusual Clinical Presentation." Case Rep Dent **2016**: 6305173.
75. De Chiara, A., et al. (2001). "Primary plasmacytoma of the breast." Arch Pathol Lab Med **125**(8): 1078-1080.
76. De Zoysa, N., et al. (2012). "Extramedullary plasmacytoma of the true vocal fold." Ear Nose Throat J **91**(8): E23-25.
77. Debnath, C. R., et al. (2015). "Primary extramedullary esophageal plasmacytoma - a case report." Mymensingh Med J **24**(2): 395-398.
78. Demetriades, N., et al. (2008). "A clinico-pathologic correlation. Extramedullary plasmacytoma." J Mass Dent Soc **57**(3): 56-58.
79. Dempewolf, R. and J. H. Lee (2008). "Extramedullary plasmacytoma presenting as a nasal mass in an immunosuppressed patient: treatment after failed primary radiotherapy." Ear Nose Throat J **87**(4): 223-225.
80. Deshpande, S. S., et al. (2014). "Extramedullary plasmacytoma in the carotid space: Expanding the differential diagnosis." Indian J Radiol Imaging **24**(4): 410-414.
81. Dhodapkar, M. V., et al. (2003). "Expression of cancer/testis (CT) antigens MAGE-A1, MAGE-A3, MAGE-A4, CT-7, and NY-ESO-1 in malignant gammopathies is heterogeneous and correlates with site, stage and risk status of disease." Cancer Immun **3**: 9.
82. Ding, W., et al., (2019). “Primary plasmablastic plasmacytoma in the stomach of an immunocompetent adult. A case report" Medicine (Baltimore) **98**(4):e14235.
83. Di Stadio, A., et al., (2020). “Extramedullary nasal plasmacytoma arising after polyp excision and the role of the inflammation in tumor development: A case report”. Mol Clin Oncol **12**(5):451-455.
84. Di Stadio, A., et al., (2021). “Septal Nasal Extramedullary Plasmacytoma: A Rare Tumor in an Unusual Area”. Ear Nose Throat J **100**(5_suppl):805S-807S.
85. Doi, A., et al. (2016). "Double Extramedullary Plasmacytoma of the Stomach with a Long-term Endoscopic Follow-up." Intern Med **55**(24): 3585-3590.
86. Doki, T., et al. (2008). "Primary isolated extramedullary plasmacytoma of the colon." Int J Colorectal Dis **23**(7): 719-720.
87. Du, Y-L. & Yan Y., (2019). “Solitary extra-medullary plasmacytoma of the true vocal cord”. Chin Med J (Engl) **132**(15):1885-1886.
88. Duletic-Nacinovic, A., et al. (2010). "Dacryocystitis provoked by recurrence of extramedullary plasmacytoma of the orbit: a case report." Tumori **96**(1): 164-167.
89. Eck, D. L., et al. (2015). "Extramedullary plasmacytoma of the small intestine." Am Surg **81**(3): E104-105.
90. Eisterer, W., et al. (2001). "CD44 isoforms are differentially regulated in plasma cell dyscrasias and CD44v9 represents a new independent prognostic parameter in multiple myeloma." Leuk Res **25**(12): 1051-1057.
91. El Maaroufi, H., et al. (2012). "Extramedullary plasmacytoma of the liver." Hematol Oncol Stem Cell Ther **5**(3): 172-173.
92. El Naderi, S., et al. (2013). "Isolated laryngeal plasmacytosis." Eur Ann Otorhinolaryngol Head Neck Dis **130**(5): 293-295.
93. Emery, J. D., et al. (1999). "Plasmacytoma of the ovary: a case report and literature review." Gynecol Oncol **73**(1): 151-154.
94. Erdogan, B. A., et al. (2013). "Extramedullary plasmacytoma of maxillary sinus." J Craniofac Surg **24**(1): e85-87.
95. Erkal, H. S., et al. (2006). "Extramedullary plasmacytoma presenting as a large lesion protruding from the nasal cavity with massive hemorrhage." J Neuroradiol **33**(2): 143-144.
96. Ersoy, O., et al. (2004). "Extramedullary plasmacytoma of the maxillary sinus." Acta Otolaryngol **124**(5): 642-644.
97. Evans, M.G., et al., (2020). “First Reported Case of Extramedullary Plasmacytoma of the Appendix”. Gastroenterology Res **13**(2):85-87.
98. Exarchakos, G., et al. (2003). "Solitary extramedullary plasmacytoma of the maxillary sinus. Case report." Acta Otorhinolaryngol Belg **57**(3): 217-220.
99. Fabbian, F., et al. (2004). "Cutaneous plasmacytoma in a hemodialysis patient." Int J Artif Organs **27**(10): 907-909.
100. Feldman, A., et al. (2015). "Solitary ovarian plasmacytoma. A case report and review of literature." Gynecol Oncol Rep **13**: 20-22.
101. Feldman, A. M., et al. (2017). "Management of gynaecologic plasmacytoma: A review article." J Obstet Gynaecol **37**(1): 5-10.
102. Feng, P. H., et al. (2008). "Solitary pleural plasmacytomas manifested as a massive pleural effusion without evidence of monoclonal gammopathy." Respirology **13**(5): 751-753.
103. Feng, Y. F., et al. (2007). "Correlation of immunophenotype of sinonasal non-Hodgkin's lymphoma to Epstein-Barr virus infection." Ai Zheng **26**(11): 1170-1176.
104. Fernandez, L. A., et al. (2001). "An unusual presentation of extramedullary plasmacytoma occurring sequentially in the testis, subcutaneous tissue, and heart." Am J Hematol **67**(3): 194-196.
105. Fernandez-Acenero, M. J., et al. (2009). "Non-epithelial lesions of the larynx: review of the 10-year experience in a tertiary Spanish hospital." Acta Otolaryngol **129**(1): 108-112.
106. Fischer, D., et al. (2007). "[Extramedullary manifestation of plasmocytoma in ENT]." Laryngorhinootologie **86**(1): 48-50.
107. Fraser, S. C., et al. (2010). "Pathology quiz case 2: extramedullary plasmacytoma (EMP)." Arch Otolaryngol Head Neck Surg **136**(6): 635, 636-637.
108. Fuchs, E. J., et al. (1998). "Chemotherapy does not nullify the ability of donor lymphocyte infusions to mediate graft-versus-host reactions." Bone Marrow Transplant **22**(3): 303-305.
109. Fujikata, S., et al. (2002). "Extramedullary plasmacytoma arising from an adrenal gland." Urology **60**(3): 514.
110. Fukuhara, S., et al. (2016). "Successful treatment of primary advanced gastric plasmacytoma using a combination of surgical resection and chemotherapy with bortezomib: A case report." Int J Surg Case Rep **27**: 133-136.
111. Gabriel, E. M. and M. Savu (2014). "Discovery of a rare ileocecal plasmacytoma." J Surg Case Rep **2014**(3).
112. Gabriel, U., et al. (2015). "Plasmacytoma of the Breast: A Report of a Rare Disease." Ethiop J Health Sci **25**(4): 373-376.
113. Galhotra, R., et al. (2012). "Primary isolated extramedullary plasmacytoma of mesentry: a rare case report." Gulf J Oncolog(12): 81-84.
114. Galieni, P., et al. (2000). "Clinical outcome of extramedullary plasmacytoma." Haematologica **85**(1): 47-51.
115. Ganesh, M., et al. (2000). "Extramedullary plasmacytoma presenting as upper back pain." J R Soc Promot Health **120**(4): 262-265.
116. Gao, B. and X. Wang (2007). "An extramedullary plasmacytoma originating from the thoracic spinal cord: magnetic resonance imaging findings. Case report." J Neurosurg Spine **6**(1): 57-59.
117. Garas, G., et al. (2010). "Extramedullary plasmacytoma of the tongue base." JRSM Short Rep **1**(7): 60.
118. Garelli, M., et al. (2011). "Imaging of a case of extramedullary solitary plasmacytoma of the trachea." Case Rep Radiol **2011**: 687203.
119. Gasz, A., et al., (2020). “Laparoscopic partial resection of retroperitoneal extramedullary plasmacytoma”. Cir Esp (Engl Ed) **98**(5):299-301.
120. Gautam, A., et al. (2017). "Extramedullary Solitary Plasmacytoma: Demonstrating the Role of (18)F-FDG PET Imaging." J Clin Diagn Res **11**(4): Xd01-xd03.
121. Ge, S., et al. (2018). "Extramedullary plasmacytoma of the larynx: Literature review and report of a case who subsequently developed acute myeloid leukemia." Oncol Lett **16**(3): 2995-3004.
122. George, S. M., et al. (2017). "Plasmacytoma as a Mimicker of Colonic Carcinoma in an Elderly Man." Case Rep Pathol **2017**: 4846018.
123. Ghatak, S., et al. (2013). "Primary solitary extramedullary plasmacytoma involving the true vocal cords in a pregnant woman." Tumori **99**(1): e14-18.
124. Ghazizadeh, M., et al. (2015). "Radioresistant Extramedullary Plasmacytoma of the Maxillary Sinus: A Case Report and review article." Iran J Otorhinolaryngol **27**(81): 313-318.
125. Ghodke, K., et al. (2015). "A retrospective study of correlation of morphologic patterns, MIB1 proliferation index, and survival analysis in 134 cases of plasmacytoma." Ann Diagn Pathol **19**(3): 117-123.
126. Gholizadeh, N., et al. (2016). "Extramedullary Plasmacytoma of the Oral Cavity in a Young Man: a Case Report." J Dent (Shiraz) **17**(2): 155-158.
127. Gianom, D., et al. (1999). "[Primary extramedullary plasmacytoma of the duodenum]." Swiss Surg **5**(1): 6-10.
128. Gibbs, J. D., et al. (2017). "A rare case of Epstein-Barr virus-related plasmacytoma involving maxillary sinus mucosa." Clin Case Rep **5**(9): 1482-1485.
129. Gilani, J. A., et al. (2011). "Plasmacytoma of the thyroid gland." J Ayub Med Coll Abbottabad **23**(4): 131-132.
130. Glasbey, J. C., et al. (2018). "Gastrointestinal manifestations of extramedullary plasmacytoma: a narrative review and illustrative case reports." Ann R Coll Surg Engl **100**(5): 371-376.
131. Gohil, M. H., et al. (2015). "Plasmacytoma rectum extending to para-rectal region." J Cancer Res Ther **11**(3): 662.
132. Gomez-Rodriguez, N., et al. (1998). "An unusual tumour of the knee: an extramedullary plasmacytoma." Br J Rheumatol **37**(6): 701-702.
133. Gonzalez-Calle, V., et al. (2016). "Multiple primary cutaneous plasmacytoma a decade after a nasal solitary extramedullary plasmacytoma: a puzzling case." Clin Case Rep **4**(12): 1096-1100.
134. Gonzalez-Garcia, J., et al. (1998). "Primary extramedullary plasmacytoma of the parotid gland: a case report and review of the literature." J Laryngol Otol **112**(2): 179-181.
135. Goussard, P., et al. (2017). "Extramedullary plasmacytoma in the airway of an HIV-positive child." Pediatr Pulmonol **52**(10): E88-e90.
136. Gouveris, H., et al. (2006). "Solitary extramedullary plasmacytoma and granulomatous sialadenitis of the parotid gland preceding a B-cell non-Hodgkin's lymphoma." Mund Kiefer Gesichtschir **10**(2): 122-125.
137. Green, J., et al., (2019). “Rare multifocal manifestation of solitary extramedullary plasmacytomas”. BMJ Case Rep. **12**(12):e232273.
138. Grover, N., et al. (2006). "Extramedullary plasmacytoma of the nasal cavity: treatment perspective in a developing nation." Ear Nose Throat J **85**(7): 434-436.
139. Guo, S. Q., et al. (2013). "Prognostic factors associated with solitary plasmacytoma." Onco Targets Ther **6**: 1659-1666.
140. Gupta, D. and J. Goyal (2015). "A rare case of extramedullary plasmacytoma arising from posterior mediastinum." Indian J Cancer **52**(3): 428-429.
141. Gupta, V., et al. (2007). "Primary isolated extramedullary plasmacytoma of colon." World J Surg Oncol **5**: 47.
142. Hackl, G., et al. (2014). "Progressive jugular vein thrombosis in cutaneous extramedullary plasmacytoma." Eur J Haematol **93**(2): 177-178.
143. Halefoglu, A. M. (2004). "Solitary extramedullary plasmacytoma arising in the pterygoid fossa." Acta Otolaryngol **124**(6): 747-750.
144. Hama, Y., et al. (2004). "Solitary extramedullary plasmacytoma of the tongue." Ulster Med J **73**(2): 132-134.
145. Han, J. H., et al. (2017). "Primary extramedullary plasmacytoma mimicking oral lichen planus." Eur J Dermatol **27**(5): 533-534.
146. Han, Y. J., et al. (2014). "Solitary extramedullary plasmacytoma in the gastrointestinal tract: report of two cases and review of literature." Korean J Gastroenterol **63**(5): 316-320.
147. Hanawa, Y., et al., (2019). “Crohn’s Disease Accompanied with Small Intestinal Extramedullary Plasmacytoma”. Intern Med **58**(14):2019-2023.
148. Handa, U., et al. (2010). "Plasma cell tumours: cytomorphological features in a series of 12 cases diagnosed on fine needle aspiration cytology." Cytopathology **21**(3): 186-190.
149. Hans, F. J., et al. (2013). "Solitary plasmacytoma presenting as an intramedullary mass of the cervical cord." J Neurol Surg A Cent Eur Neurosurg **74 Suppl 1**: e13-17.
150. Harada, S., et al. (2014). "Magnifying narrow-band imaging endoscopy for the diagnosis of gastric primary extramedullary plasmacytoma: a first case report." Endoscopy **46 Suppl 1 UCTN**: E435-436.
151. Hari, C. K. and D. G. Roblin (2000). "Solitary plasmacytoma of the parotid gland." Int J Clin Pract **54**(3): 197-198.
152. Hashiguchi, K., et al. (2004). "Extramedullary plasmacytoma of the rectum arising in ulcerative colitis: case report and review." Gastrointest Endosc **59**(2): 304-307.
153. Hassan, M. J., et al. (2014). "Extramedullary plasmacytoma of the thyroid: report of a rare case." Blood Res **49**(4): 280-283.
154. Hayes-Lattin, B., et al. (2003). "Pulmonary and intracerebral plasmacytomas in a patient without multiple myeloma: a case report." Am J Hematol **73**(2): 131-134.
155. Hazarika, P., et al. (2011). "Solitary extramedullary plasmacytoma of the sinonasal region." Indian J Otolaryngol Head Neck Surg **63**(Suppl 1): 33-35.
156. He, J., et al. (2015). "Irradiation combined with surgery for function preservation in the treatment of extramedullary plasmacytoma of the left labia majora during pregnancy: A case report." Oncol Lett **10**(5): 3238-3242.
157. He, L., et al., (2020). “Epstein-Barr virus-positive plasmacytoma in immunocompetent patients: a diagnostic dilemma”. Int J Clin Exp Pathol **13**(3):582-586.
158. Heo, D., et al. (2015). "A Case of Posterior Mediastinal Plasmacytoma Confounded by Community-Acquired Pneumonia." Tuberc Respir Dis (Seoul) **78**(3): 262-266.
159. Hidaka, H., et al. (2000). "A case of extramedullary plasmacytoma arising from the nasal septum." J Laryngol Otol **114**(1): 53-55.
160. Him, M., et al. (2017). "Rare Presentation of Primary Extramedullary Plasmacytoma as Lip Lesion." Case Rep Oncol Med **2017**: 4296802.
161. Honavar, S. G., et al. (2001). "Extramedullary plasmacytoma confined to the choroid." Am J Ophthalmol **131**(2): 277-278.
162. Hong, W., et al. (2009). "Solitary extramedullary plasmacytoma in retroperitoneum: a case report and review of the literature." World J Gastroenterol **15**(19): 2425-2427.
163. Hotz, M. A., et al. (1999). "Extramedullary solitary plasmacytoma of the head and neck. A clinicopathological study." Ann Otol Rhinol Laryngol **108**(5): 495-500.
164. Huoh, K. C., et al. (2011). "Extramedullary plasmacytoma of the tonsil." Case Rep Otolaryngol **2011**: 430809.
165. Husaric, S., et al. (2013). "Solitary extramedullary plasmocytoma of the liver." Acta Med Acad **42**(1): 85-86.
166. Hussong, J. W., et al. (1999). "Extramedullary plasmacytoma. A form of marginal zone cell lymphoma?" Am J Clin Pathol **111**(1): 111-116.
167. Hwang, D. W., et al. (2010). "Primary hematolymphoid malignancies involving the extrahepatic bile duct or gallbladder." Leuk Lymphoma **51**(7): 1278-1287.
168. Ibe, W., et al. (1999). "A nonhealing ulcer diagnosed as extramedullary plasmocytoma of the limb eight years after cardiac transplantation." Transplantation **68**(6): 901-904.
169. Ignjatovic, M., et al. (2016). "Solitary extramedullary plasmacytoma of the duodenum and pancreas: A case report and review of the literature." Vojnosanit Pregl **73**(4): 402-407.
170. Ioannidis, O., et al. (2010). "Giant cutaneous plasmacytoma of the sacrococcygeal region." J Cutan Med Surg **14**(2): 90-94.
171. Iseri, M., et al. (2009). "Synchronous presentation of extramedullary plasmacytoma in the nasopharynx and the larynx." Ear Nose Throat J **88**(11): E9-12.
172. Ishtiaq, R., et al. (2018). "Nasal plasmacytoma: a rare cause of persistent epistaxis." Postgrad Med **130**(6): 507-510.
173. Jantunen, E., et al. (2005). "Autologous stem cell transplantation in patients with high-risk plasmacytoma." Eur J Haematol **74**(5): 402-406.
174. Jiang, J., et al. (2012). "An unusual occurrence of solitary extramedullary plasmacytoma in the conjunctiva." Oncol Lett **4**(2): 245-246.
175. Jones, J. E., et al. (2008). "Primary extramedullary plasmacytomas of the colon." Am Surg **74**(9): 873-874.
176. Joseph, A. A., et al. (2015). "Extramedullary plasmacytoma: an unusual neoplasm in a HIV-positive patient." Int J STD AIDS.
177. Kahara, T., et al. (2001). "Extramedullary plasmacytoma in the adrenal incidentaloma." Clin Endocrinol (Oxf) **55**(2): 267-270.
178. Kakarlapudi, V. V., et al. (1999). "Extramedullary plasmacytoma in the anterior cervical region." Otolaryngol Head Neck Surg **121**(3): 296-297.
179. Kalan, A., et al. (2000). "Solitary extramedullary plasmacytoma of tonsil - a rare location." Indian J Otolaryngol Head Neck Surg **52**(3): 285-289.
180. Kamijo, T., et al. (2002). "A case of extramedullary plasmacytoma of the larynx." Acta Otolaryngol Suppl(547): 104-106.
181. Kamisawa, H., et al. (2008). "Testicular metastasis from an extramedullary plasmacytoma." Int J Clin Oncol **13**(5): 464-466.
182. Kang, D. Y., et al. (2016). "Successful treatment of a primary gastric plasmacytoma mimicking intractable gastric ulcer by using high-dose dexamethasone therapy: a case report." J Med Case Rep **10**: 79.
183. Kanthan, R. and B. Torkian (2003). "Solitary Plasmacytoma of the Parotid Gland with Crystalline Inclusions: A Case Report." World J Surg Oncol **1**(1): 12.
184. Karakullukcu, B., et al. (2011). "Surgical debulking combined with photodynamic therapy to manage residual extramedullary plasmacytoma of the nasopharynx." Photodiagnosis Photodyn Ther **8**(3): 264-266.
185. Katayama, Y., et al., (2020). “Extramedullary Plasmacytoma of the Stomach“. Clin Gastroenterol Hepatol **18**(8):e91-e92.
186. Katodritou, E., et al. (2008). "Successful treatment of extramedullary gastric plasmacytoma with the combination of bortezomib and dexamethasone: first reported case." Leuk Res **32**(2): 339-341.
187. Kaviani, A., et al. (2004). "Recurrence of primary extramedullary plasmacytoma in breast both simulating primary breast carcinoma." World J Surg Oncol **2**: 29.
188. Khaliq, W., et al. (2010). "Solitary extramedullary plasmacytoma of the bladder: a case report and literature." Oncology (Williston Park) **24**(9): 832-835.
189. Khankirawatana, B. and W. L. Ginete (2004). "Primary extramedullary plasmacytoma of the heart." Clin Cardiol **27**(6): 368.
190. Khayyata, S., et al. (2007). "Retroperitoneal extramedullary anaplastic plasmacytoma masquerading as sarcoma: Report of a case with an unusual presentation and imprint smears." Acta Cytol **51**(3): 434-436.
191. Kim, K. S., et al. (2012). "Solitary Extramedullary Plasmacytoma of the Apex of Arytenoid: Endoscopic, CT, and Pathologic Findings." Clin Exp Otorhinolaryngol **5**(2): 107-111.
192. Kitamura, F., et al. (2018). "Primary extramedullary plasmacytoma of the sigmoid colon with perforation: a case report." Surg Case Rep **4**(1): 28.
193. Klein, T., et al. (2010). "The first description of an extramedullary plasmacytoma of the ureter." Urol Int **84**(1): 122-124.
194. Knecht, P., et al. (2008). "Rapid progressive extramedullary plasmacytoma in the orbit." Klin Monbl Augenheilkd **225**(5): 514-516.
195. Kodani, T., et al. (2011). "Successful endoscopic mucosal resection of a solitary extramedullary plasmacytoma in the sigmoid colon." Endoscopy **43 Suppl 2 UCTN**: E298-299.
196. Koletsa, T., et al. (2012). "A case of a primary cutaneous plasmacytoma presenting in adolescence." Am J Dermatopathol **34**(5): 537-540.
197. Kraus-Tiefenbacher, U., et al. (2004). "Plasmocytoma of the urethra." Onkologie **27**(2): 166-168.
198. Krause, S., et al. (2011). "Radiotherapy of solitary plasmacytoma." Ann Hematol **90**(9): 1093-1097.
199. Krebs, S., et al. (2017). "Solitary Extramedullary Plasmacytoma of the Cricoid Cartilage-Case Report." Front Oncol **7**: 284.
200. Kremer, M., et al. (2005). "Primary extramedullary plasmacytoma and multiple myeloma: phenotypic differences revealed by immunohistochemical analysis." J Pathol **205**(1): 92-101.
201. Krishnamoorthy, N., et al. (2010). "A rare case of primary gastric plasmacytoma: an unforeseen surprise." J Cancer Res Ther **6**(4): 549-551.
202. Kulkarni, R., et al., (2020). “Persistent orofacial pain associated with nasopharyngeal extramedullary plasmacytoma”. Spec Care Dentist **40**(5):519-524.
203. Kumar, A., et al. (2015). "Solitary extramedullary plasmacytoma of thoracic epidural space presenting with dorsal compressive myelopathy: A case report and review of literature." J Neurosci Rural Pract **6**(3): 410-412.
204. Kupfer, T. J., et al. (2011). "[Persistent extramedullary plasmacytoma of the nose after radiotherapy]." HNO **59**(1): 119-121.
205. Kusunoki, T., et al. (2007). "Extramedullary plasmacytoma of the larynx: a case report from Japan." Ear Nose Throat J **86**(12): 763-764.
206. Kuyumcu, G., et al. (2017). "Unusual Cause of Sphenoidal Sinus Mass: Extramedullary Plasmacytoma." Can J Neurol Sci **44**(3): 324-325.
207. Lai, C. R., et al. (2010). "A solitary pelvic extramedullary plasmacytoma." South Med J **103**(8): 831-833.
208. Lai, C-Y., et al., (2020). “Clinical Features of Head and Neck Solitary Extramedullary Plasmacytoma in Taiwan”. In Vivo **34**(1):261-265.
209. Lao, W., et al. (2007). "Solitary extramedullary plasmacytoma of anorectum." Int J Colorectal Dis **22**(9): 1117-1118.
210. Laszlo, I., et al. (2009). "Synchronous myeloproliferative and inflammatory disease of the nasal cavity and paranasal sinuses: an interesting differential diagnostic problem." Rhinology **47**(3): 323-326.
211. Lattuneddu, A., et al. (2004). "A case of primary extramedullary plasmacytoma of the colon." Int J Colorectal Dis **19**(3): 289-291.
212. Lazarevic, V., et al. (2001). "Diffuse primary plasmacytoma of the lung." Haematologia (Budap) **31**(2): 161-165.
213. Lee, C. H., et al. (2014). "Liquid-based cytologic findings of solitary extramedullary plasmacytoma in thyroid: a case report identified with fine-needle aspiration cytology." Diagn Cytopathol **42**(11): 964-969.
214. Lee, K. S., et al. (2004). "Solitary dural extramedullary plasmacytoma with inv(9)(p13q21)." Am J Clin Oncol **27**(6): 638-639.
215. Lee, S. H., et al. (2013). "Primary Isolated Extramedullary Plasmacytoma in the Colon." Gastroenterology Res **6**(4): 152-155.
216. Lee, S. Y., et al. (2005). "A case of extramedullary plasmacytoma arising from the posterior mediastinum." Korean J Intern Med **20**(2): 173-176.
217. LeNoir, B., et al., (2019). ”Extramedullary Plasmacytoma of the Right Main Bronchus“. Ann Thorac Surg. **108**(2):e119-e120.
218. Lewis, K., et al. (2007). "Extramedullary plasmacytomas of the larynx and parapharyngeal space: imaging and pathologic features." Ear Nose Throat J **86**(9): 567-569.
219. Li, Y., et al. (2007). "Extramedullary plasmacytoma involving the bilateral adrenal glands on MR imaging." Korean J Radiol **8**(3): 246-248.
220. Li, Y., et al., (2019). “Extramedullary plasmacytoma of the kidney in an HIV-positive patient. A case report”. Medicine (Baltimore) **98**(52):e18422.
221. Liao, L. J., et al. (2006). "Pathology quiz case 2. Extramedullary plasmacytoma (EMP) of the nasopharynx." Arch Otolaryngol Head Neck Surg **132**(11): 1269, 1271.
222. Liebross, R. H., et al. (1999). "Clinical course of solitary extramedullary plasmacytoma." Radiother Oncol **52**(3): 245-249.
223. Lim, C. C., et al. (2013). "Extramedullary plasmacytoma of the eyelid: a case report and review of literature." Clin Exp Optom **96**(3): 349-351.
224. Lim, Y. H., et al. (2005). "A case of primary plasmacytoma of lymph nodes." Korean J Intern Med **20**(2): 183-186.
225. Liu, H. Y., et al. (2010). "Prognosis and expression of lambda light chains in solitary extramedullary plasmacytoma of the head and neck: two case reports and a literature review." J Int Med Res **38**(1): 282-288.
226. Liu, M. L., et al. (2006). "Chronic idiopathic myelofibrosis terminating in extramedullary anaplastic plasmacytoma." Leuk Lymphoma **47**(2): 315-322.
227. Loghavi, S., et al. (2015). "Epstein-Barr virus-positive plasmacytoma in immunocompetent patients." Histopathology **67**(2): 225-234.
228. Loong, S.P., et al., (2019). ”Solitary Extramedullary Plasmacytoma of the Sphenoid Sinus: A Case Report” Indian J Otolaryngol Head Neck Surg **71**(Suppl 3):1692-1694.
229. Lomeo, P. E., et al. (2007). "Extramedullary plasmacytoma of the nasal sinus cavities." Am J Otolaryngol **28**(1): 50-51.
230. Lopes da Silva, R. (2012). "Pancreatic involvement by plasma cell neoplasms." J Gastrointest Cancer **43**(2): 157-167.
231. Lopes da Silva, R. (2012). "Extramedullary plasmacytoma of the small intestine: clinical features, diagnosis and treatment." J Dig Dis **13**(1): 10-18.
232. Lou, Y., et al. (2005). "A patient of extramedullary cutaneous and gingival plasmacytomas." Eur J Haematol **75**(2): 171.
233. Loyo, M., et al. (2013). "Plasmacytoma of the larynx." Am J Otolaryngol **34**(2): 172-175.
234. Lu, G. & Zhang, Q., (2020). “Extramedullary Plasmacytoma of False Vocal Cord: Case Report” Ear Nose Throat J 145561320971929.
235. Lu, T., et al. (2017). "Primary pancreatic plasmacytoma: a rare case report." BMC Gastroenterol **17**(1): 167.
236. Luh, S. P., et al. (2007). "Extramedullary plasmacytoma (EMP): Report of a case manifested as a mediastinal mass and multiple pulmonary nodules and review of literature." World J Surg Oncol **5**: 123.
237. Maheshwari, G. K., et al. (2001). "Extramedullary plasmacytoma of the larynx: a case report." J Indian Med Assoc **99**(5): 267-268.
238. Maheshwari, R. and S. Maheshwari (2009). "Extramedullary pasmacytoma masquerading as chalazion." Orbit **28**(2-3): 191-193.
239. Mainali, N. R., et al. (2014). "A case of disseminated recurrent retroperitoneal plasmacytoma with left obstructive hydronephrosis." BMJ Case Rep **2014**.
240. Majadob, K., et al. (2013). "Recurrence of extramedullary plasmacytoma of the breast." Ecancermedicalscience **7**: 322.
241. Majerovic, M., et al. (2012). "Extramedullary plasmacytoma imitating neoplasm of the gallbladder fossa after cholecystectomy." Coll Antropol **36**(1): 331-333.
242. Majumdar, S., et al. (2002). "Solitary plasmacytoma and extramedullary plasmacytoma of the paranasal sinuses and soft palate." J Laryngol Otol **116**(11): 962-965.
243. Makis, W., et al. (2012). "Gastric recurrence of a primary colon plasmacytoma: staging and evaluating response to therapy with 18F-FDG PET/CT." Br J Radiol **85**(1009): e4-9.
244. Manabe, M., et al. (2010). "Extramedullary plasmacytoma of the dura mimicking meningioma." Int J Hematol **91**(5): 731-732.
245. Mandagere, K. A., et al. (1998). "An unusual sellar mass--solitary plasmacytoma." Endocr Pract **4**(6): 382-386.
246. Manganaris, A., et al. (2010). "Uncommon presentation of nasopharyngeal extramedullary plasmacytoma: a case report and literature review." B-ENT **6**(2): 143-146.
247. Mann, G., et al. (2007). "Extramedullary plasmacytoma of the adenoids." Pediatr Blood Cancer **48**(3): 361-362.
248. Marchiori, E., et al. (2010). "[Extramedullary plasmacytoma that simulates pulmonary metastasis]." Arch Bronconeumol **46**(8): 445-446.
249. Marom, T., et al. (2009). "Clinical photograph. Sinonasal extramedullary plasmacytoma." Otolaryngol Head Neck Surg **141**(4): 533-534.
250. Martin, P. J., et al. (2011). "Extranodal testicular anaplastic versus plasmablastic plasma cell tumor: A rare case with diagnostic dilemma in a developing country." Indian J Med Paediatr Oncol **32**(1): 49-54.
251. Mei, Y. H., et al. (2017). "An extramedullary plasmacytoma in the kidney of a 14-year-old girl: Case report and review of the literature." Medicine (Baltimore) **96**(6): e6092.
252. Mendenhall, W. M., et al. (2003). "Solitary plasmacytoma of bone and soft tissues." Am J Otolaryngol **24**(6): 395-399.
253. Menon, S., et al. (2014). "Dual pathology of the submandibular gland: plasmacytoma and pleomorphic adenoma." BMJ Case Rep **2014**.
254. Mertens de Wilmars, M., et al. (2015). "Solitary extramedullary plasmocytoma of the thyroid: a case report and histological approach to plasma cells infiltrate in the thyroid gland." Acta Clin Belg **70**(2): 133-137.
255. Merza, H. and R. Sarkar (2016). "Solitary extraosseous plasmacytoma." Clin Case Rep **4**(9): 851-854.
256. Meziane, M., et al. (2012). "Solitary plasmocytoma: ghost tumour?" Int J Oral Maxillofac Surg **41**(1): 17-19.
257. Michalaki, V. J., et al. (2003). "Definitive radiotherapy for extramedullary plasmacytomas of the head and neck." Br J Radiol **76**(910): 738-741.
258. Micozkadioglu, S. D., et al. (2009). "Extramedullary plasmacytoma of the septum." B-ENT **5**(3): 169-171.
259. Miller, F. R., et al. (1998). "Plasmacytomas of the head and neck." Otolaryngol Head Neck Surg **119**(6): 614-618.
260. Mimura, R., et al. (2010). "Extramedullary plasmacytoma involving perirenal space accompanied by extramedullary hematopoiesis and amyloid deposition." Jpn J Radiol **28**(4): 309-313.
261. Mirarchi, M., et al. (2016). "Successful surgical resection of solitary plasmacytoma of the liver mimicking hepatocellular carcinoma. A case report." Ann Ital Chir **87**: 343-349.
262. Mitropoulou, G., et al. (2017). "Solitary Plasmacytoma of the Mesentery: A Systematic Clinician's Diagnosis." Case Rep Oncol Med **2017**: 5901503.
263. Miwa, W., et al. (2019). "Solitary extramedullary plasmacytoma of the rectum complicating ulcerative colitis." Clin J Gastroenterol **12**(2): 160-165.
264. Mjoli, M., et al. (2016). "Solitary extramedullary plasmacytoma of the colon, rectum and anus." S Afr J Surg **54**(2): 45-47.
265. Mohammad Taheri, Z., et al. (2010). "Primary pulmonary plasmacytoma with diffuse alveolar consolidation: a case report." Patholog Res Int **2010**: 463465.
266. Mokhtar, G. A., et al. (2006). "Cytopathology of extramedullary plasmacytoma of the bladder: a case report." Acta Cytol **50**(3): 339-343.
267. Momiyama, N., et al. (1999). "Extramedullary Plasmacytoma of the Breast with Serum IgD Monoclonal Protein: A Case Report and Review of the Literature." Breast Cancer **6**(3): 217-221.
268. Mondal, S. K., et al. (2015). "Primary extramedullary plasmacytoma of ovary: Report of a rare neoplasm." J Cancer Res Ther **11**(4): 923-924.
269. Mongha, R., et al. (2010). "Plasmacytoma of the kidney." Saudi J Kidney Dis Transpl **21**(5): 931-934.
270. Morariu, I., et al. (2011). "Triple manifestation of extramedullary plasmacytoma in the upper airway: an unusual clinical entity." J Laryngol Otol **125**(9): 970-972.
271. Morinaga, R., et al. (2010). "Extramedullary plasmacytoma as an uncommon cause of gastrorrhagia." Intern Med **49**(15): 1679-1680.
272. Moriyama, H., et al. (2006). "Primary extramedullary plasmacytoma of the small intestine. A case report and review of the literature." J Exp Clin Cancer Res **25**(1): 129-134.
273. Moshref, M., et al. (2007). "Extramedullary plasmacytoma of the gingiva." Arch Iran Med **10**(1): 91-93.
274. Murthy, V., et al. (2003). "Solitary extramedullary plasmacytoma of the premaxilla." Australas Radiol **47**(2): 202-204.
275. Nagai, T., et al. (2017). "Extramedullary plasmacytoma of the ureter in an HIV-positive patient." Int Cancer Conf J **6**(4): 171-174.
276. Nagasaka, T., et al. (2001). "Localized amyloidosis and extramedullary plasmacytoma involving the larynx of a child." Hum Pathol **32**(1): 132-134.
277. Nair, S. K., et al. (2014). "Extramedullary plasmacytoma of gingiva and soft tissue in neck." J Clin Diagn Res **8**(11): ZD16-18.
278. Nakagawa, Y., et al. (2011). "Minute primary extramedullary plasmacytomas of the large intestine." Endoscopy **43 Suppl 2 UCTN**: E105-106.
279. Nakashima, T., et al. (2006). "Extramedullary plasmacytoma of the larynx." Auris Nasus Larynx **33**(2): 219-222.
280. Nakaya, A., et al., (2020). “Retrospective analysis of plasmacytoma in Kansai Myeloma Forum Registry” Int J Hematol **112**(5):666-673.
281. Nanda, K. D., et al. (2012). "Plasmacytoma masquerading as an abscess." J Investig Clin Dent **3**(3): 236-239.
282. Naqash, S., et al. (2015). "An unusual breast malignancy." Gulf J Oncolog **1**(18): 7-9.
283. Naqash, S., et al. (2015). "Fungating extramedullary plasmocytoma of breast; a unique case presentation." Indian J Cancer **52**(1): 43-44.
284. Narayanan, G., et al. (2016). "Bilateral synchronous plasmacytoma of the testis." Proc (Bayl Univ Med Cent) **29**(2): 196-197.
285. Narumi, T., et al. (2005). "CT and MRI findings of a solitary extramedullary plasmacytoma of the oropharynx: case report." Radiat Med **23**(8): 574-577.
286. Nie, S., et al. (2016). "Primary pulmonary plasmacytoma: a case report introduction." World J Surg Oncol **14**(1): 205.
287. Nikolidakis, A. A., et al. (2000). "Solitary extramedullary plasmacytoma of the nasopharynx." J Otolaryngol **29**(4): 254-257.
288. Nowak-Sadzikowska, J. and M. Weiss (1998). "Extramedullary plasmacytoma of the larynx. Analysis of 5 cases." Eur J Cancer **34**(9): 1468.
289. Oishi, T., et al. (2006). "Extramedullary plasmacytoma extensively affecting the sella turcica and paranasal sinuses." Clin Neuropathol **25**(1): 44-47.
290. Oka, K., et al. (2010). "Aggressive and fatal extramedullary plasmacytoma: a report of two cases." APMIS **118**(5): 418-420.
291. Okamura, S., et al. (2005). "Jejunal extramedullary plasmacytoma." Gastrointest Endosc **61**(1): 107-108.
292. Oliveira, R. C., et al. (2017). "Primary gastric plasmacytoma: a rare entity." BMJ Case Rep **2017**.
293. Onal, C., et al. (2012). "Primary solitary extramedullary plasmacytoma of the tongue." Ear Nose Throat J **91**(7): 292-295.
294. Oriakhi, M., et al., (2019). “Endobronchial Obstruction by a Solitary Extramedullary Plasmacytoma With Light-chain Amyloidosis”. J Bronchology Interv Pulmonol **26**(2):e24-e26.
295. Orme, C. M., et al. (2012). "Clinically amyopathic dermatomyositis in a patient with an extramedullary plasmacytoma of the tongue." J Dermatol **39**(7): 656-657.
296. Ornstein, D. L., et al. (2002). "Nonmyeloablative allogeneic peripheral blood stem cell transplantation for multifocal extramedullary plasmacytomas progressing after autologous transplantation." Bone Marrow Transplant **29**(1): 71-74.
297. Ozdemir, R., et al. (2005). "Plasmacytoma of the hard palate." J Craniofac Surg **16**(1): 164-169.
298. Ozdemir, S., et al. (2013). "A case of extramedullary plasmacytoma in the sphenoid sinus with unilateral loss of vision." J Craniomaxillofac Surg **41**(2): 140-143.
299. Ozkok, A., et al. (2012). "An unusual case of renal failure due to solitary plasmacytoma: parenchymal invasion of the kidney." Ren Fail **34**(5): 640-642.
300. Ozoner, B., et al. (2018). "Solitary Extramedullary Plasmacytoma Mimicking Acute Subdural Hematoma." World Neurosurg **120**: 521-524.
301. Pacheco, T. R., et al. (2003). "Extramedullary plasmacytoma in cardiac transplant recipients." J Am Acad Dermatol **49**(5 Suppl): S255-258.
302. Padhi, P. & El-Behery, R., (2020). “Extramedullary Solitary Plasmacytoma with Anaplastic Features of the Nasopharynx”. Case Rep Hematol **2020**:8845546.
303. Paksoy, N. (2010). "Cervical lymph node metastasis of extramedullary plasmacytoma of the tonsil presenting with granulomatous lymphadenitis in fine needle aspiration cytology." Acta Cytol **54**(5): 733-736.
304. Palacios, E., et al. (2002). "Extramedullary plasmacytoma in the nasal cavity." Ear Nose Throat J **81**(8): 499-500.
305. Pantelidou, D., et al. (2005). "Extramedullary plasmacytoma: report of two cases with uncommon presentation." Ann Hematol **84**(3): 188-191.
306. Papadaki, H. A., et al. (2000). "Epstein-Barr virus-associated high-grade anaplastic plasmacytoma in a renal transplant patient." Leuk Lymphoma **36**(3-4): 411-415.
307. Park, B. J., et al. (2010). "Disseminated plasmacytoma of the thyroid." Ear Nose Throat J **89**(3): 137-139.
308. Park, C. H., et al. (2009). "Treatment of solitary extramedullary plasmacytoma of the stomach with endoscopic submucosal dissection." Gut Liver **3**(4): 334-337.
309. Park, J. I., et al., (2021). “A rare case of primary solitary endobronchial plasmacytoma”. Thorac Cancer **12**(6):958-961.
310. Park, S. H., et al. (2009). "Endoscopic endonasal transsphenoidal resection of solitary extramedullary plasmacytoma in the sphenoid sinus with destruction of skull base." J Korean Neurosurg Soc **46**(2): 156-160.
311. Park, S. Y., et al. (2014). "Successful treatment of a gastric plasmacytoma using a combination of endoscopic submucosal dissection and oral thalidomide." Clin Endosc **47**(6): 564-567.
312. Park, Y. M. (2016). "Imaging Findings of Plasmacytoma of Both Breasts as a Preceding Manifestation of Multiple Myeloma." Case Rep Med **2016**: 6595610.
313. Paul, R. H., et al. (2000). "Neuropsychological and magnetic resonance imaging abnormalities associated with a plasmacytoma of the frontal dura: a case report." Neuropsychiatry Neuropsychol Behav Neurol **13**(2): 143-147.
314. Petrucci, M. T., et al. (2003). "Extramedullary liver plasmacytoma a rare presentation." Leuk Lymphoma **44**(6): 1075-1076.
315. Pichi, B., et al. (2011). "Cricoid-based extramedullary plasmocytoma." J Craniofac Surg **22**(6): 2361-2363.
316. Pierre-Filho Pde, T., et al. (2004). "Orbital extramedullary plasmacytoma leading to exenteration." Can J Ophthalmol **39**(5): 557-559.
317. Pino, M., et al. (2015). "Extramedullary Plasmacytoma of the Larynx Treated by a Surgical Endoscopic Approach and Radiotherapy." Case Rep Otolaryngol **2015**: 951583.
318. Pinto, J. A., et al. (2012). "Extramedullary plasmacytoma of the larynx." Int Arch Otorhinolaryngol **16**(3): 410-413.
319. Pitini, V., et al. (2008). "Extramedullary plasmacytoma presented as a non-functional invasive pituitary macro-adenoma." J Neurooncol **88**(2): 227-229.
320. Ponniah, I. and S. Rajan (2012). "Plasmablastic extramedullary plasmacytoma." Indian J Pathol Microbiol **55**(1): 104-106.
321. Pratibha, C. B., et al. (2009). "Plasmacytoma of larynx--a case report." J Voice **23**(6): 735-738.
322. Rai, S. P., et al. (2003). "Solitary tracheal plasmacytoma." Indian J Chest Dis Allied Sci **45**(4): 269-272.
323. Rakover, Y., et al. (2000). "Isolated extramedullary plasmacytoma of the true vocal fold." J Laryngol Otol **114**(7): 540-542.
324. Ramadan, A., et al. (2000). "Testicular plasmacytoma in a patient with the acquired immunodeficiency syndrome." Tumori **86**(6): 480-482.
325. Ramirez-Anguiano, J., et al. (2012). "Extramedullary plasmacytoma of the larynx: a case report of subglottic localization." Case Rep Otolaryngol **2012**: 437264.
326. Reddi, V. R., et al. (1998). "Primary plasmacytoma of testis. Report of a case." Indian J Cancer **35**(4): 152-155.
327. Reed, V., et al. (2011). "Solitary plasmacytomas: outcome and prognostic factors after definitive radiation therapy." Cancer **117**(19): 4468-4474.
328. Refai, F., et al., (2019). “A Case Report of Thyroid Plasmacytoma and Literature Update”. J Microsc Ultrastruct. **8**(2):75-79.
329. Reyhan, M., et al. (2005). "Sonographic diagnosis of a tracheal extramedullary plasmacytoma." J Ultrasound Med **24**(7): 1031-1034.
330. Ridal, M., et al. (2012). "Solitary extramedullary plasmacytoma of the thyroid gland." Case Rep Otolaryngol **2012**: 282784.
331. Rimmer, C., et al. (2013). "Esophageal plasmacytoma diagnosed in a patient presenting with cardiac symptoms: a novel case." Case Rep Gastrointest Med **2013**: 121670.
332. Rogers, C. G., et al. (2004). "Extraosseous (extramedullary) plasmacytoma of the adrenal gland." Arch Pathol Lab Med **128**(7): e86-88.
333. Roumy, A., et al. (2013). "Localized amyloid light-chain amyloidosis and extramedullary plasmacytoma of the mitral valve." Ann Thorac Surg **95**(5): 1782-1784.
334. Ruiz Santiago, F., et al. (2010). "Soft tissue extramedullary plasmacytoma." Case Rep Med **2010**: 307902.
335. Rutherford, K., et al. (2009). "Extramedullary plasmacytoma of the larynx in an adolescent: a case report and review of the literature." Ear Nose Throat J **88**(2): E1-7.
336. Sabir, H., et al. (2015). "A 65-year old female with synchronous HIV and Extramedullary Plasmacytoma of Maxillary sinus." Gulf J Oncolog **1**(19): 18-23.
337. Sachdev, R., et al., (2019). “Extramedullary plasmacytoma of tonsil: an unusual site”. Indian J Pathol Microbiol **62**(1):167-168.
338. Sakai, Y., et al. (2008). "Extramedullary plasmacytoma of the tonsil diagnosed by fine-needle aspiration cytology." Ann Diagn Pathol **12**(1): 41-43.
339. Sakiyama, S., et al. (2005). "Extramedullary plasmacytoma immunoglobulin D (lambda) in the chest wall and the subglottic region." J Thorac Cardiovasc Surg **129**(5): 1168-1169.
340. Sarin, H., et al. (2009). "Extramedullary plasmacytoma, a report of five cases diagnosed by FNAC." Cytopathology **20**(5): 328-331.
341. Scarberry, K., et al. (2014). "Solitary extramedullary plasmacytoma of the penis." Urol Ann **6**(3): 242-243.
342. Schneider, I., et al. (2014). "Pseudo-Popeye syndrome: extramedullary plasmacytoma manifesting in skeletal muscle." Neurology **82**(6): 544-545.
343. Schor, A. P., et al. (2010). "Primary plasmacytoma of the cervix in a 21-year-old female patient." Int J Gynecol Pathol **29**(3): 290-293.
344. Schulze, B., et al. (2009). "[Solitary extramedullary plasmocytoma of the conjunctiva]." Ophthalmologe **106**(2): 149-151.
345. Schwartz, T. H., et al. (2001). "Association between intracranial plasmacytoma and multiple myeloma: clinicopathological outcome study." Neurosurgery **49**(5): 1039-1044; discussion 1044-1035.
346. Seidl, R. O., et al. (1999). "[Metastasizing oropharyngeal tumor. Extramedullary plasmacytoma]." HNO **47**(12): 1070-1071.
347. Selvan, H., et al., (2020). “Trabeculectomy in an unsuspected extramedullary iris–ciliary body plasmacytoma”. Eur J Ophthalmol 2020:1120672120920225.
348. Seoane, J., et al. (1999). "Primary extramedullary plasmacytoma of the palate." Otolaryngol Head Neck Surg **120**(4): 530.
349. Shah, C., et al. (2001). "Extramedullary plasmacytoma of the submandibular gland." J Laryngol Otol **115**(12): 1023-1025.
350. Shahrizal, T. A., et al. (2009). "Isolated extramedullary plasmacytoma of the middle turbinate." Ear Nose Throat J **88**(2): 786-789.
351. Shao, H., et al. (2010). "Nodal and extranodal plasmacytomas expressing immunoglobulin a: an indolent lymphoproliferative disorder with a low risk of clinical progression." Am J Surg Pathol **34**(10): 1425-1435.
352. Sharma, L. M., et al. (2004). "Retro-peritoneal plasmacytoma: a case report and review of literature." Indian J Cancer **41**(3): 133-134.
353. Sheth, S., et al. (2013). "(18)F-fluorodeoxyglucose positron emission tomography/computed tomography imaging of extra-medullary plasmacytoma of the ovary." Indian J Nucl Med **28**(3): 185-186.
354. Shetty, P., et al. (2017). "Oral extramedullary plasmacytoma in a HIV positive patient." Oral Oncol **64**: e4-e5.
355. Shields, C. L., et al. (2007). "Sequential bilateral solitary extramedullary plasmacytoma of the ciliary body." Cornea **26**(6): 759-761.
356. Shimokihara, K., et al. (2018). "Extramedullary plasmacytoma of the testis: A case report." Urol Case Rep **16**: 101-103.
357. Shreif, J. A., et al. (2001). "Extramedullary plasmacytoma of the nasal cavity." Otolaryngol Head Neck Surg **124**(1): 119-120.
358. Shuke, N., et al. (2001). "Accumulation of Tc-99m HMDP in extramedullary plasmacytoma of the stomach." Clin Nucl Med **26**(4): 354-355.
359. Shukla, A., et al. (2011). "Extramedullary plasmacytoma presenting as a mediastinal mass." Turk J Haematol **28**(3): 228-231.
360. Sia, D. I., et al. (2010). "Extramedullary plasmacytoma arising from the lacrimal gland." Clin Experiment Ophthalmol **38**(9): 895-898.
361. Sidlo, J. and H. Sidlova (2019). "Sudden and unexpected death due to intracranial sellar extramedullary plasmacytoma." J Forensic Leg Med **61**: 89-91.
362. Skerget, M., et al., (2020). “Surgery results in low relapse and progression rates in extramedullary plasmacytoma of the head and neck: A case cohort and review of the literature”. Hematol Rep **12**(2):8396.
363. Sodhi, K. S., et al. (2013). "Solitary extramedullary plasmacytoma of the nasal tract: an unusual cause of epistaxis." Ear Nose Throat J **92**(6): E51.
364. Soni, N. K., et al. (2002). "Solitary extramedullary plasmacytoma - larynx." Indian J Otolaryngol Head Neck Surg **54**(4): 309-310.
365. Spence, R. A., et al. (2013). "Primary plasmacytoma of the kidney." Case Rep Urol **2013**: 239580.
366. Stein, J., et al. (2016). "Primary Urethral Plasmacytoma Treated with High-Dose-Rate Brachytherapy: A Case Report." Urol Int **97**(3): 369-372.
367. Stevic, R., et al. (2018). "Rare tracheal tumor: Solitary plasmacytoma." J Postgrad Med **64**(2): 115-118.
368. Straetmans, J. and R. Stokroos (2008). "Extramedullary plasmacytomas in the head and neck region." Eur Arch Otorhinolaryngol **265**(11): 1417-1423.
369. Strojan, P., et al. (2002). "Extramedullary plasmacytoma: clinical and histopathologic study." Int J Radiat Oncol Biol Phys **53**(3): 692-701.
370. Suh, Y. G., et al. (2012). "Radiotherapy for solitary plasmacytoma of bone and soft tissue: outcomes and prognostic factors." Ann Hematol **91**(11): 1785-1793.
371. Sukumaran, R., et al. (2014). "Extramedullary plasmacytoma of the trachea." Head Neck Pathol **8**(2): 220-224.
372. Sulzner, S. E., et al. (1998). "Extramedullary plasmacytoma of the head and neck." Am J Otolaryngol **19**(3): 203-208.
373. Sun, N., et al. (2012). "A case of extramedullary solitary plasmacytoma arising at the uterine cervix." Eur J Gynaecol Oncol **33**(4): 423-424.
374. Suska, A., et al., (2019). “A rare case of a solitary extramedullary plasmacytoma of the palatine tonsil”. Pol Arch Intern Med. **129**(3):201-203.
375. Takahashi, R., et al. (2005). "Plasmacytoma of the urinary bladder in a renal transplant recipient." Int J Hematol **81**(3): 255-257.
376. Takahashi, T., et al. (2016). "Primary Extramedullary Plasmacytoma in the Gastroduodenal Canal Associated With Epstein-Barr Virus-Associated Adenocarcinoma of the Stomach: A Case Report." Int J Surg Pathol **24**(8): 757-762.
377. Tamiolakis, D., et al. (2003). "Fine-needle aspiration cytology of a para-aortical solitary plasma-cell tumor." Leuk Lymphoma **44**(10): 1831-1833.
378. Tan, J., et al. (2012). "Complete remission of localised gastric plasmacytomas following definitive radiotherapy." J Med Imaging Radiat Oncol **56**(3): 328-331.
379. Tang, R-R., et al., (2019) Multiple extramedullary plasmacytomas of the trachea and pharyngeal tissue: a case report and literature review". Onco Targets Ther **12**:1433-1437.
380. Tani, E., et al. (1999). "Fine-needle aspiration cytology and immunocytochemistry of soft-tissue extramedullary plasma-cell neoplasms." Diagn Cytopathol **20**(3): 120-124.
381. Templeton, K. J., et al. (2001). "Extramedullary plasmacytoma of the hand: a case report." J Hand Surg Am **26**(4): 781-785.
382. Tenzel, P. A., et al. (2017). "Extramedullary plasmacytoma of the lateral rectus muscle." Orbit **36**(2): 78-80.
383. Tojima, I., et al. (2012). "Endoscopic resection of malignant sinonasal tumours with or without chemotherapy and radiotherapy." J Laryngol Otol **126**(10): 1027-1032.
384. Tokatli, F., et al. (2008). "Extramedullary plasmacytoma: clinicopathology, immunohistochemistry and therapeutic approach to a case with a tonsillar site." Hematol Oncol Stem Cell Ther **1**(4): 241-245.
385. Tomita, Y., et al. (1998). "Plasmacytoma of the gastrointestinal tract in Korea: higher incidence than in Japan and Epstein-Barr virus association." Oncology **55**(1): 27-32.
386. Tournier-Rangeard, L., et al. (2006). "Radiotherapy for solitary extramedullary plasmacytoma in the head-and-neck region: A dose greater than 45 Gy to the target volume improves the local control." Int J Radiat Oncol Biol Phys **64**(4): 1013-1017.
387. Townend, P. J., et al. (2017). "Bilateral extramedullary adrenal plasmacytoma: case report and review of the literature." Int J Endocr Oncol **4**(2): 67-73.
388. Treglia, G., et al. (2014). "An Unusual Case of Extramedullary Plasmacytoma of the Hypopharynx Detected by (18)F-FDG PET/CT." Nucl Med Mol Imaging **48**(4): 328-329.
389. Tristano, A. G. (2004). "Extramedullary orbital and cutaneous plasmacytomas." Am J Hematol **77**(2): 203-204.
390. Trivedi, S., et al. (2016). "Extramedullary plasmacytoma of the gingiva." BMJ Case Rep **2016**.
391. Tsang, D. S., et al. (2016). "Treatment and outcomes for primary cutaneous extramedullary plasmacytoma: a case series." Curr Oncol **23**(6): e630-e646.
392. Tsaur, I., et al. (2010). "[Gross haematuria as the primary symptom of extramedullary IgM plasmacytoma of the bladder]." Urologe A **49**(7): 850-854.
393. Tzen, C. Y., et al. (2006). "Is extramedullary plasmacytoma an oligoclonal tumour with clonal selection during tumour progression?" Histopathology **48**(4): 469-471.
394. Uceda-Montanes, A., et al. (2000). "Extramedullary plasmacytoma of the orbit." Acta Ophthalmol Scand **78**(5): 601-603.
395. Ucmak, D., et al. (2014). "Multiple primary cutaneous plasmacytomas: an unusual presentation." J Cutan Med Surg **18**(5): 361-364.
396. Ujiie, H., et al. (2012). "A case of primary solitary pulmonary plasmacytoma." Ann Thorac Cardiovasc Surg **18**(3): 239-242.
397. Ustun, M. O., et al. (2001). "Extramedullary plasmacytoma of the parotid gland. Report of a case with extensive amyloid deposition masking the cytologic and histopathologic picture." Acta Cytol **45**(3): 449-453.
398. Vallisa, D., et al. (1998). "Extramedullary plasmacytoma in a patient with AIDS: report of a case and review of the literature." Tumori **84**(4): 511-514.
399. Vanan, I., et al. (2009). "Solitary extramedullary plasmacytoma of the vocal cord in an adolescent." J Clin Oncol **27**(35): e244-247.
400. Velez, D., et al. (2007). "Laryngeal plasmacytoma presenting as amyloid tumour: a case report." Eur Arch Otorhinolaryngol **264**(8): 959-961.
401. Vera-Alvarez, J., et al. (2003). "Extramedullary plasmacytoma presenting as a primary mass in the breast. A case report." Acta Cytol **47**(6): 1107-1110.
402. Verim, A., et al. (2014). "Extramedullary plasmacytoma of the frontal sinus: case report and Turkish literature review." Turk J Haematol **31**(3): 301-306.
403. Vosmik, M., et al. (2007). "Solitary extramedullary plasmacytoma in the oropharynx: advantages of intensity-modulated radiation therapy." Clin Lymphoma Myeloma **7**(6): 434-437.
404. Vrettou, A. R., et al. (2014). "Cardiac plasmacytoma: a rare clinical entity." Tex Heart Inst J **41**(5): 554-557.
405. Wan, X., et al. (2001). "Primary extramedullary plasmacytoma in the atria of the heart." Cardiovasc Pathol **10**(3): 137-139.
406. Wang, J., et al. (2018). "Retroperitoneal extramedullary plasmacytoma: A case report and review of the literature." Medicine (Baltimore) **97**(46): e13281.
407. Wang, J., et al. (1999). "Metastatic extramedullary plasmacytoma of the lung." Leuk Lymphoma **35**(3-4): 423-425.
408. Wang, M., et al. (2015). "Extramedullary plasmacytoma of the cricoid cartilage progressing to multiple myeloma: A case report." Oncol Lett **9**(4): 1764-1766.
409. Wang, N-N., et al., (2019). “A rare solitary duodenum plasmacytoma”. Rev Esp Enferm Dig **111**(7):563.
410. Wang, Y., et al. (2013). "Primary extramedullary plasmacytoma of the penis: a case report." Jpn J Clin Oncol **43**(10): 1030-1033.
411. Wang, Y., et al. (2015). "Computed tomography findings of a solitary extramedullary plasmacytoma of the spleen: A case report and literature review." Oncol Lett **9**(1): 219-222.
412. Watanabe, N., et al. (2000). "A case of retroperitoneal extramedullary plasmacytoma with multiple metastases." Clin Imaging **24**(6): 365-367.
413. Webb, C. J., et al. (2002). "Primary extramedullary plasmacytoma of the tongue base. Case report and review of the literature." ORL J Otorhinolaryngol Relat Spec **64**(4): 278-280.
414. Weber, A., et al. (2002). "[Parapharyngeal space-occupying lesions. Differential diagnosis based on case examples]." HNO **50**(3): 223-229.
415. Wei, J. Y., et al. (2009). "Bortezomib in treatment of extramedullary plasmacytoma of the pancreas." Hepatobiliary Pancreat Dis Int **8**(3): 329-331.
416. Wei, S., et al. (2012). "Primary endobronchial plasmacytoma involving local lymph nodes and presenting with rare immunoglobulin G lambda monoclonal gammopathy." Can Respir J **19**(3): e28-30.
417. Wijaya, J. and D. McHarg (2001). "Ga-67 and Tl-201 scintigraphy in extramedullary plasmacytoma: a case report." Clin Nucl Med **26**(7): 596-598.
418. Windfuhr, J. P. and G. Ott (2002). "Extramedullary plasmacytoma manifesting as a palpable mass in the nasal cavity." Ear Nose Throat J **81**(2): 110-114.
419. Wise, J. N., et al. (2001). "Primary pulmonary plasmacytoma: a case report." Chest **120**(4): 1405-1407.
420. Woo Park, C., et al. (2013). "Solitary extramedullary plasmacytoma presenting as an endobronchial mass." Intern Med **52**(18): 2113-2116.
421. Wu, W., et al. (2013). "Extraosseous plasmacytoma with an aggressive course occurring solely in the CNS." Neuropathology **33**(3): 320-323.
422. Wyeth, A., et al., (2020). “Primary Plasma Cell Neoplasm of the Kidney Without Formation of a Mass and Its Renal Manifestations: An Interstitial Variant of Renal Plasmacytoma?” Clin Lymphoma Myeloma Leuk **20**(9):e551-e555.
423. Xiang, P., et al., (2019). ”Pancreatic tumor in type 1 autoimmune pancreatitis: a diagnostic challenge”. BMC Cancer 19(1):814.
424. Xing, Y., et al. (2015). "Prognostic factors of laryngeal solitary extramedullary plasmacytoma: a case report and review of literature." Int J Clin Exp Pathol **8**(3): 2415-2435.
425. Yan, J., et al. (2017). "Solitary plasmacytoma associated with Epstein-Barr virus: a clinicopathologic, cytogenetic study and literature review." Ann Diagn Pathol **27**: 1-6.
426. Yao, C. M., et al. (2012). "Solitary extramedullary plasmacytomas of the thyroid in Hashimoto's thyroiditis." Thyroid **22**(8): 861-862.
427. Yavas, O., et al. (2004). "Extramedullary plasmacytoma of nasopharynx and larynx: synchronous presentation." Am J Hematol **75**(4): 264-265.
428. Yoshida, A., et al. (2008). "Incidental detection of concurrent extramedullary plasmacytoma and amyloidoma of the nasopharynx on [18F]fluorodeoxyglucose positron emission tomography/computed tomography." J Clin Oncol **26**(35): 5817-5819.
429. Yoshida, T., et al. (2004). "Biclonal extramedullary plasmacytoma arising in the peritoneal cavity: report of a case." Surg Today **34**(4): 379-382.
430. Yumori, J. W., et al. (2010). "Conjunctival plasmacytoma." Optometry **81**(5): 234-239.
431. Zappacosta, R., et al. (2012). "Solitary plasmacytoma of the tonsillar site associated with actinomyces infection: the possible role of IL-6." J Biol Regul Homeost Agents **26**(3): 571-575.
432. Zazpe, I., et al. (2007). "Solitary thoracic intradural extramedullary plasmacytoma." Acta Neurochir (Wien) **149**(5): 529-532; discussion 532.
433. Zehlicke, T. and L. Gramer (2002). "[Extramedullary plasmocytoma of the left tonsil. A rare cause of tonsillar asymmetry]." HNO **50**(3): 240-243.
434. Zeiser, R., et al. (2004). "Extramedullary plasmocytoma with local amyloidosis presenting as a lump on the lip." Br J Haematol **125**(6): 679.
435. Zeng, Z., et al. (2012). "Successful bortezomib treatment in combination with dexamethasone and thalidomide for previously untreated epidural plasmacytoma." Oncol Lett **3**(3): 557-559.
436. Zhang, H., et al. (2011). "Complete resection of a mediastinal solitary extramedullary plasmacytoma and reconstruction of right pulmonary artery and superior vena cava." Ann Thorac Surg **92**(6): 2244-2246.
437. Zhang, L., et al. (2016). "18F-FDG PET/CT Metabolic Activity in a Patient With Solitary Extramedullary Plasmacytoma of the Lung." Clin Nucl Med **41**(3): 232-234.
438. Zhang, S. Q., et al. (2013). "Renal plasmacytoma: Report of a rare case and review of the literature." Oncol Lett **5**(6): 1839-1843.
439. Zhang, X., et al. (2018). "Extramedullary plasmacytoma of the trachea: A case report and review of the literature." Medicine (Baltimore) **97**(3): e9594.
440. Zhang, Y., et al. (2015). "A frontal extramedullary plasmacytoma mimicking parafalcine meningioma." J Craniofac Surg **26**(2): e188-190.
441. Zhang, Y., et al. (2015). "Extramedullary plasmacytoma associated with an ectopic tooth in the nasal cavity." Ear Nose Throat J **94**(10-11): E43-46.
442. Zhao, Z. H., et al. (2014). "Imaging findings of primary gastric plasmacytoma: a case report." World J Gastroenterol **20**(29): 10202-10207.
443. Zhou, Y., et al. (2012). "Treatment of primary isolated extramedullary plasmacytoma of esophagus with endoscopic submucosal dissection." Clin Gastroenterol Hepatol **10**(3): e21-22.
444. Zhu, X., et al., (2021). “Extramedullary Plasmacytoma: Long-Term Clinical Outcomes in a Single-Center in China and Literature Review”. Ear Nose Throat J **100**(4):227-232.
445. Zhu, Z., et al. (2017). "Incomplete Colonic Obstruction Caused by Extramedullary Plasmacytoma." Clin Gastroenterol Hepatol **15**(3): e69-e70.
446. Zuo, Z., et al. (2011). "Extraosseous (extramedullary) plasmacytomas: a clinicopathologic and immunophenotypic study of 32 Chinese cases." Diagn Pathol **6**: 123.
